# Thyroxine Induces Acute Relaxation of Rat Skeletal Muscle Arteries via Integrin αvβ3, ERK1/2 and Integrin-Linked Kinase

**DOI:** 10.3389/fphys.2021.726354

**Published:** 2021-09-14

**Authors:** Ekaterina K. Selivanova, Dina K. Gaynullina, Olga S. Tarasova

**Affiliations:** ^1^Department of Human and Animal Physiology, Faculty of Biology, M.V. Lomonosov Moscow State University, Moscow, Russia; ^2^Department of Physiology, Pirogov Russian National Research Medical University, Moscow, Russia; ^3^Laboratory of Exercise Physiology, Institute of Biomedical Problems, Russian Academy of Sciences, Moscow, Russia

**Keywords:** integrin αvβ3, integrin-linked kinase, non-genomic effect, sural artery, thyroxine, smooth muscle

## Abstract

**Aim:** Hyperthyroidism is associated with a decreased peripheral vascular resistance, which could be caused by the vasodilator genomic or non-genomic effects of thyroid hormones (TH). Non-genomic, or acute, effects develop within several minutes and involve a wide tissue-specific spectrum of molecular pathways poorly studied in vasculature. We aimed to investigate the mechanisms of acute effects of TH on rat skeletal muscle arteries.

**Methods:** Sural arteries from male Wistar rats were used for isometric force recording (wire myography) and phosphorylated protein content measurement (Western blotting).

**Results:** Both triiodothyronine (T3) and thyroxine (T4) reduced contractile response of sural arteries to α_1_-adrenoceptor agonist methoxamine. The effect of T4 was more prominent than T3 and not affected by iopanoic acid, an inhibitor of deiodinase 2. Endothelium denudation abolished the effect of T3, but not T4. Integrin αvβ3 inhibitor tetrac abolished the effect of T4 in endothelium-denuded arteries. T4 weakened methoxamine-induced elevation of phospho-MLC2 (Ser19) content in arterial samples. The effect of T4 in endothelium-denuded arteries was abolished by inhibiting ERK1/2 activation with U0126 as well as by ILK inhibitor Cpd22 but persisted in the presence of Src- or Rho-kinase inhibitors (PP2 and Y27632, respectively).

**Conclusion:** Acute non-genomic relaxation of sural arteries induced by T3 is endothelium-dependent and that induced by T4 is endothelium-independent. The effect of T4 on α_1_-adrenergic contraction is stronger compared to T3 and involves the suppression of extracellular matrix signaling via integrin αvβ3, ERK1/2 and ILK with subsequent decrease of MLC2 (Ser19) phosphorylation.

## Introduction

Thyroid hormones (TH), triiodothyronine (T3), and thyroxine (T4), play an essential role in the regulation of the vascular system (Epstein et al., 2001). This becomes evident in patients with hyperthyroidism demonstrating a decrease in peripheral vascular resistance (Vargas et al., 2006; Danzi and Klein, 2014). Although this connection between vascular resistance and thyroid status is well known, the mechanisms behind it are poorly understood. A decrease in peripheral vascular resistance may be due to increased tissue vascularization and/or a reduction of vascular tone. Both acute and chronic experimental hyperthyroidism could lead to decrease of the contractile response to the α_1_-adrenergic agonist (McAllister et al., 1998; Honda et al., 2000) and increase in relaxatory response to the acetylcholine (McAllister et al., 1998; Honda et al., 2000; Bussemaker et al., 2003; Iwata and Honda, 2004). Apparently, TH-induced alterations in the vascular reactivity could substantially contribute to the drop of peripheral vascular resistance. Nevertheless, the molecular mechanisms mediating the effects of TH observed in acute and chronic hyperthyroidism are not fully investigated.

TH may act via two different mechanisms: genomic and non-genomic (Axelband et al., 2010; Davis et al., 2015; Selivanova and Tarasova, 2020). During the canonical genomic action, TH are forming complexes with thyroid hormone receptors TRα and TRβ in the nucleus with T3 demonstrating higher affinity to these receptors than T4 (Schroeder et al., 2014). Hormone-receptor complexes act as transcription factors for the target genes (Williams, 2000; Paquette et al., 2011). All other TH effects that are not associated with direct regulation of gene expression by nuclear TRα and TRβ are attributed to the non-genomic action (Cheng et al., 2010; Flamant et al., 2017). Since non-genomic actions are not necessarily dependent on transcription (Cao et al., 2005), they could occur in shorter periods (within several minutes) than genomic and, therefore, may be referred to as rapid or acute (Hiroi et al., 2006). Previous studies in mice have revealed the relevance of non-genomic effects of TH *in vivo* for hippocampal synapses maturation (Martin et al., 2014) as well as maintenance of heart rate, body temperature, blood glucose, and triglyceride concentration (Hönes et al., 2017). Presumably, both genomic and non-genomic action of thyroid hormones contribute to the decrease of peripheral resistance in hyperthyroidism.

Non-genomic effects may contribute to the decrease of peripheral vascular resistance by vasorelaxation. It has been shown that both T3 and T4 can induce rapid non-genomic dilation of arteries (Yoneda et al., 1998). Such effects of TH were observed in mice (Gachkar et al., 2019), rats (Lozano-Cuenca et al., 2016), hamsters (Colantuoni et al., 2005), rabbits (Ishikawa et al., 1989), and humans (Krasner et al., 1997) but the data on involved signaling pathways are rather controversial. Some studies reported that T3 induced more prominent acute vasodilation than T4 (Park et al., 1997; Aoki et al., 2015), some studies—that the effects of T3 and T4 are similar (Yoneda et al., 1998) or that T4 is more potent than T3 (Zwaveling et al., 1997). Several studies confirmed that acute non-genomic vasodilation is endothelium-dependent (Lozano-Cuenca et al., 2016) but others showed that it is not (Carrillo-Sepulveda et al., 2010; Cai et al., 2015). This diversity of data could be related to the large number of receptors mediating the non-genomic effects of TH and heterogeneity in the expression of these receptors among different vascular beds.

Non-genomic effects of TH may be mediated by various receptors including cytoplasmic TRα and TRβ, truncated isoforms of TRα and integrin αvβ3 (Plateroti et al., 2001; Hiroi et al., 2006; Kalyanaraman et al., 2014; Selivanova and Tarasova, 2020; Davis et al., 2021; Geist et al., 2021). TH binding to these receptors could initiate different signaling pathways involving Src-kinase, phosphoinositide-3 kinase (PI3K)/Akt cascade, extracellular signal-regulated protein kinases (ERK1/2) (Cao et al., 2005; Moeller et al., 2005; Lin et al., 2009). TH-induced activation of this cascades may lead to the shuttling of nuclear thyroid hormone receptors (Lin et al., 2009), mammalian target of rapamycin complex (mTOR) (Cao et al., 2005), signal transducer and activator of transcription (STAT1α, STAT3) (Lin et al., 1999b) and other factors to the nucleus with subsequent modulation of target genes expression, e.g., hypoxia-induced factor-1α (Lin et al., 2009). Of note, all these pathways have been discovered in studies on the cell cultures like human glioma U-87 MG cells and human skin fibroblasts, not in arteries.

Most research groups focused on the mechanisms of T3-induced acute vasodilation (Colantuoni et al., 2005; Hiroi et al., 2006; Cai et al., 2015; Lozano-Cuenca et al., 2016). Hiroi et al. (2006) were able to elegantly show that in endothelial cell culture T3 can form complex with cytoplasmic TRα1 and p85α, the regulatory subunit of PI3K, with subsequent activation of Akt and endothelial NO-synthase (eNOS) (Hiroi et al., 2006). Noteworthy, the molecular mechanisms of T4-induced acute vasodilation remain to be investigated. The effect of T4 could be no less physiologically relevant than the effect of T3 since in non-genomic action T4 is considered as an active hormone because of higher affinity compared to T3 to some receptors, e.g., to S2 site of integrin αvβ3 (Lin et al., 2009, 2018). For instance, stimulation of angiogenesis is predominantly induced by T4 and mediated by integrin αvβ3 (Bergh et al., 2005; Balzan et al., 2013; Liu et al., 2014). In addition, blood content of T4 is more than ten times higher compared to T3 content (Quesada et al., 2002; Ghasemi et al., 2013; Gaynullina et al., 2018a).

This study aimed at the mechanisms of acute effects of TH on rat skeletal muscle arteries. The skeletal muscle vascular bed is the thyroid-dependent region since the blood flow in skeletal muscle is significantly changed both in hyperthyroid humans (Frey, 1967; Martin et al., 1992) and animals with experimental hyperthyroidism (McAllister et al., 1995; Bausch and McAllister, 2003). However, the intracellular signaling pathways responsible for the vasorelaxatory effects of TH and the relative contribution of T3 and T4 remain rather unexplored.

## Materials and Methods

### Animals

All experimental procedures in this study were conformed to the European Convention on the protection of animals used for scientific purposes (EU Directive 2010/63/EU) and approved by the Biomedical Ethics Committee of the Russian Federation State Research Center Institute for Biomedical Problems, Russian Academy of Sciences (protocol 472, approval date May 29, 2018). Adult male Wistar rats (body weight 300–400 g) were obtained from the vivarium of the Institute of General Pathology and Pathophysiology (Moscow, Russia) and then kept in the laboratory animal unit of the Biological Faculty of Lomonosov Moscow State University till the day of sacrifice. Rats were decapitated under CO_2_ narcosis.

### Wire Myography

Sural (gastrocnemius feed) arteries were isolated in physiological salt solution I (PSS I, for composition hereinafter see section “Solutions”), cut into 2 mm long segments and mounted in the wire myograph chamber (410A or 620M, DMT A/S, Denmark) for isometric force recording. Then PSS I was changed to PSS II which was warmed to 37°C and bubbled with gas mixture 5% CO_2_ + 95% O_2_ to maintain pH 7.4. Transducer readings were continuously recorded at 10 Hz sampling rate using E14-140 analogue-to-digital data converter (LCard, Russia) and PowerGraph 3.3 software (DISoft, Russia). After heating the PSS II was changed to the Ca-free PSS (preheated to 37°C) to prevent contraction during the normalization procedure. After normalization, the segments were set to 0.9 d100, where d100 is the inner diameter of the fully relaxed vessel exposed to the transmural pressure of 100 mmHg (Mulvany and Halpern, 1977). After that, the Ca-free PSS was changed back to PSS II, which was used till the end of experiment. Then the arteries were activated by noradrenaline (10 μM) and α_1_-adrenoceptor agonist methoxamine (MX, 10 μM), each contraction was followed by a washout period (at least 15 min). In some experiments (as indicated in text and figure legends), the endothelium was removed with a rat whisker (after segment mounting, before heating). Endothelium integrity or removal was confirmed by the presence of at least 70% relaxatory response or absence of relaxatory response to acetylcholine (10 μM) applied on the top of MX-induced contraction (1–3 μM).

**FIGURE 1:**
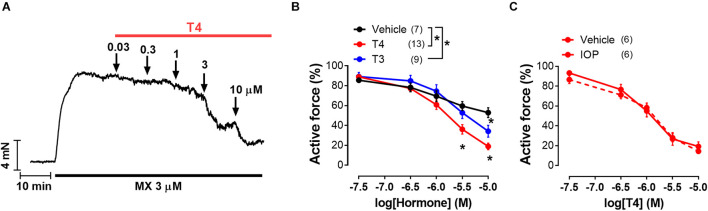
T4 induces more pronounced vasorelaxation of rat sural arteries than T3. **(A)** Original recording of the T4-induced concentration-dependent vasodilation of the sural artery precontracted by the α_1_-adrenoceptor agonist methoxamine (MX). **(B)** Concentration-response relationships to thyroid hormones of the methoxamine-precontracted sural arteries. **(C)** Concentration-response relationships to T4 of sural arteries precontracted by methoxamine in the presence of 5′-deiodinase inhibitor iopanoic acid (IOP, 100 μM) or its vehicle. For panels **(B,C)** average active force value for 10-min interval before addition of the first hormone concentration was taken as 100%. Number in parentheses represents the number of animals. *p < 0.05 compared to vehicle group (Repeated Measures ANOVA with Tukey’s *post hoc* test).

Two experimental protocols were used. In the first one, 25 min after the activation procedure, the arteries were exposed to the cumulative concentration–response relationship (CRR) to MX applied in concentrations 0.01–3 μM (3 min each), then 10–100 μM (2 min each). This relationship was performed to confirm similar reactivity of segments to MX and to determine their maximal active force. Thirty min later, MX was applied (1–5 μM) to induce submaximal contraction (60–70% of maximal active force in CRR1). After contraction reached the plateau, the cumulative CRR to the thyroid hormone (T3 or T4, concentration range 0.03–10 μM) or an equivalent volume of vehicle (DMSO, time-control segments) was performed (Figure 1A). In some experiments, a 5′-deiodinase inhibitor or equivalent vehicle volume was added to the chambers for 30 min before the CRR to T4. T3 and T4 were applied in 10 min intervals which are too short to mediate genomic effects but sufficient to trigger the non-genomic effects according to studies on endothelial and smooth muscle cell cultures (Ojamaa et al., 1996; Hiroi et al., 2006). The total CRR time was about 1 h.

For inhibitory analysis of molecular mechanisms behind observed relaxatory effects we used experimental protocol with two consecutive CRRs to MX. In both CRRs MX was applied in concentrations 0.01–3 μM (3 min each), then 10–100 μM (2 min each). As in the first protocol, the first CRR was needed to prove similar initial responses of segments to MX. After first CRR, the segments were incubated with a hormone, an inhibitor or respective vehicle during 20–30 min (time sufficient for the full manifestation of T3 and T4 effects, the exact duration depended on the inhibitor used, see section “Drugs”) and then second CRR was performed.

**FIGURE 2:**
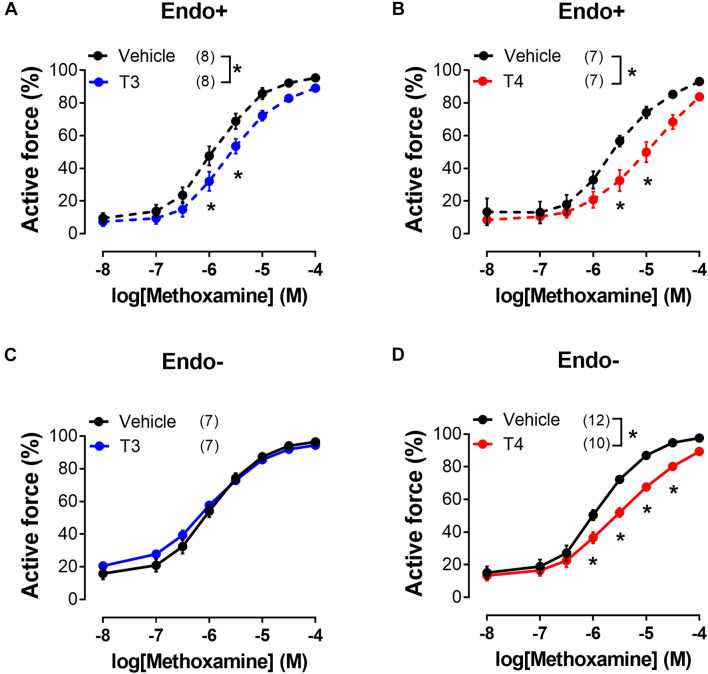
T3 and T4 weaken contractile responses of sural arteries to methoxamine via endothelium-dependent and endothelium-independent mechanisms, respectively. Concentration-response relationships of endothelium-intact (Endo+) **(A,B)** and endothelium-denuded (Endo-) **(C,D)** arteries to methoxamine in the presence of vehicle or T3 **(A,C)** or T4 **(B,D)**. Number in parentheses represents the number of animals. *p < 0.05 compared to vehicle group (Repeated Measures ANOVA with Tukey’s *post hoc* test).

**FIGURE 3:**
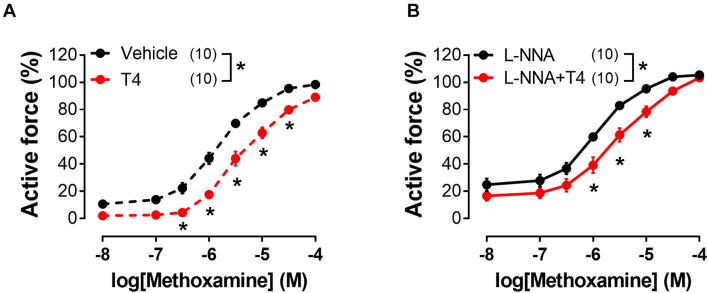
T4-induced suppression of contractile responses of sural arteries to methoxamine persists in the presence of nitric oxide synthase inhibitor L-NNA. Concentration-response relationships to methoxamine obtained after preincubation with T4 or vehicle in the absence **(A)** or presence of L-NNA **(B)** (100 μM). Number in parentheses represents the number of animals. *p < 0.05 compared to respective control group (Repeated Measures ANOVA with Tukey’s *post hoc* test).

**FIGURE 4:**
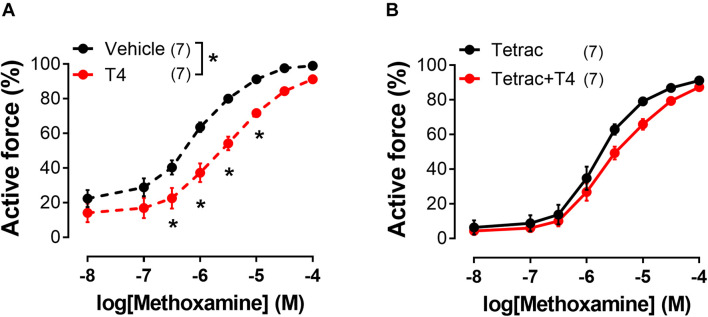
Integrin αvβ3 inhibitor tetrac abolishes T4-induced suppression of contractile responses of endothelium-denuded sural arteries to methoxamine. Concentration-response relationships to methoxamine obtained after preincubation with T4 or vehicle in the absence **(A)** or presence **(B)** of tetrac (3 μM). Number in parentheses represents the number of animals. *p < 0.05 compared to vehicle group (Repeated Measures ANOVA with Tukey’s *post hoc* test).

During the analysis of wire myography results, we calculated active force by subtracting the passive force value (level of force in the fully relaxed preparations, obtained in the Ca^2+^-free solution) from all recorded values (before each CRR and at each hormone or MX concentration). All active force values in hormone-induced CRRs were expressed as a percentage of active force value in MX-induced precontraction (shown in Figure 1). All active force values in MX-induced CRRs were expressed as a percentage of the maximum active force value recorded in the respective first CRR (second CRRs are shown in Figures 2–4, 6, 7). To estimate the maximum response (E_max_) and sensitivity of arteries to contractile agonists (by EC_50_, the concentration which causes half-maximum response), individual concentration–response relationships were fitted to a sigmoidal function in GraphPad Prizm 7.0 (La Jolla, CA, United States). We also calculated the area under the curve (AUC) in GraphPad Prism 7.0 to compare the overall effects of T4 under different conditions. AUC was expressed as a percentage of a mean value in respective control segments.

### Phosphorylated Protein Expression Levels

Sural arteries were cut in 10-mm long segments and mounted in the analogue of the wire myograph system. To determine protein phosphorylation, arterial segments were subjected to similar protocols as in contraction experiments. Briefly, the preparations were stretched to 0.9 d100 and activated by noradrenaline (10 μM) and MX (10 μM). Then the preparations were incubated with T4 (10 μM) or vehicle (5 μl DMSO) for 30 min. After incubation, some segments were exposed to MX (concentration range 0.01–10 μM) and some—to the same volume of H_2_O. Right after that, the preparations were immediately frozen in 15% trichloroacetic acid/acetone/dry ice slurry and then stored for 3 h in dry ice. Then the preparations were transferred into acetone for 15 min, after which they were allowed to dry in empty tubes. Each sample consisted of two 10-mm long segments from the same rat. In addition, we collected two reference samples of small mesenteric arteries, one of which was exposed to the MX (10 μM), and the other was not. These samples were present on each membrane and used for cross-membrane data analysis (see below).

The arterial samples were homogenized in SDS-buffer (for composition see section Solutions), centrifuged at 14,000 *g* for 2 min and heated at 99°C for 2 min; the supernatant was kept at −20°C. Proteins were separated by SDS-PAGE and transferred to nitrocellulose membrane (Santa Cruz Biotechnology, United States) using a Trans-Blot Turbo transfer system (Bio-Rad, United States). The transfer was visualized with Ponceau S stain and the membrane was cut into three parts at the level of 48 and 28 kDa protein marker (Thermo Fisher Scientific, United States). All parts were blocked with 5% non-fat milk (Applichem, Germany) TBS-T and then incubated overnight (membrane parts are listed from the bottom to top):

1) with antibodies against phospho-MLC2-Ser19 (3675S, Cell Signaling, mouse, 1:2,000 in TBS-T with 5% milk, Sigma, United States);
2) with antibodies against β-actin (4970S, Cell Signaling, rabbit, 1:2,000 in TBS-T with 5% milk, Sigma, United States);
3) with antibodies against phospho-Akt-Ser473 (9271S, Cell Signaling, rabbit, 1:1,000 in TBS, Sigma, United States).

After that, all membranes were exposed to 1 h incubation with appropriate secondary antibodies: anti-mouse (7076S, Cell Signaling, 1:5,000) or anti-rabbit (7074S, Cell Signaling, 1:10,000) in 5% milk and visualized with Super Signal West Dura Substrate (Thermo Fisher Scientific, United States) using ChemiDoc (Bio-Rad, United States). Western blotting experiments were analyzed in ImageLab Software 6.0 (Bio-Rad, United States). The phosphorylated protein of interest to β-actin ratio was calculated in each sample. The average ratio in two reference samples of mesenteric arteries was taken as 100%.

### Solutions

1. Physiological salt solution for vessel isolation (PSS I), in mM: 145 NaCl, 4.5 KCl, 1.2 NaH_2_PO_4_, 1 MgSO_4_, 0.1 CaCl_2_, 0.025 EDTA, 5 HEPES.
2. PSS for myograph experiments (PSS II), in mM: 120 NaCl, 26 NaHCO_3_, 4.5 KCl, 1.2 NaH_2_PO_4_, 1.0 MgSO_4_, 1.6 CaCl_2_, 5.5 D-glucose, 0.025 EDTA, 5 HEPES; equilibrated with 5% CO_2_ in 95% O_2_.
3. Ca-free PSS, in mM: 120 NaCl, 26 NaHCO_3_, 4.5 KCl, 1.2 NaH_2_PO_4_, 1.0 MgSO_4_, 5.5 D-glucose, 0.1 EGTA, 5 HEPES; equilibrated with 5% CO_2_ in 95% O_2_.
4. SDS-buffer: 0.0625 M Tris-HCl (pH 6.8), 2.5% SDS, 10% water-free glycerin, 2.47% dithiothreitol, 0.002% bromophenol blue supplemented with protease and phosphatase inhibitors (aprotinin 50 mg/ml, leupeptin 100 mg/ml, pepstatin 30 mg/ml, NaF 2 mg/ml, and Na_3_VO_4_ 180 mg/ml).
5. TBS: 50 mM Tris-HCl, 150 mM NaCl, pH 7.6.
6. TBS-T: TBS with 0.1% Tween.

### Drugs

Noradrenaline, acetylcholine, methoxamine (all dissolved in H_2_O), as well as T3, T4, iopanoic acid, tetrac (all dissolved in DMSO), were obtained from Sigma (United States). N^ω^ -Nitro-L-arginine (L-NNA, dissolved in H_2_O) was obtained from ALEXIS Biochemicals (United States). U0124, U0126, and compound22 (all dissolved in DMSO) were obtained from Calbiochem (United States). PP3 and PP2 (both dissolved in DMSO) were obtained from ApexBio (United States). Y27632 (dissolved in H_2_O) was obtained from Tocris (United Kingdom). Incubation time with inhibitors used in second protocol (two CRRs to MX) was 20 min (U0124, U0126, Y27632, PP2, PP3) or 30 min (tetrac, compound22). Thyroid hormone was added to the myograph chamber right after the inhibitor or vehicle. Inhibitor concentrations were selected according to the literature data: 100 μM IOP (Kaplan and Yaskoski, 1980; Coppola et al., 2005), 100 μM L-NNA (Gaynullina et al., 2013), 3 μM tetrac (Meng et al., 2011; Zanatta et al., 2013), 10 μM U0126 (Streefkerk et al., 2004), 10 μM compound22 (Lee et al., 2011), 10 μM PP2 (Zavaritskaya et al., 2017), 3 μM Y27632 (Gaynullina et al., 2018b; Mochalov et al., 2018). The total concentration of DMSO in the myograph chamber did not exceed 0.7% (v/v) in the first protocol (CRR to thyroid hormones) and 0.2% (v/v) in the second protocol.

### Statistical Data Analysis

Statistical analysis was performed in GraphPad Prism 7.0. The normality of the data distribution was confirmed using the Shapiro–Wilk test. Unpaired Student’s *t*-test, one-way ANOVA or Repeated Measures ANOVA were used, as appropriate. The level of statistically significant differences was set as *p* = 0.05. All data are given as mean ± SEM; *n* represents the number of animals.

## Results

### Acute Effects of T3 and T4 on Arteries

Both T4 and T3 were able to induce concentration-dependent relaxation of sural arteries precontracted by α_1_-adrenoceptor agonist MX (Figures 1A,B). Importantly, T4 induced more prominent relaxation than T3 (Figure 1B). The minimal effective concentrations of T4 and T3 were 3 and 10 μM, respectively (Figure 1B). In time-control experiments (vehicle group), we observed spontaneous tone decline: at the end of the test the contraction decreased to 52.9 ± 4.3% from the precontraction level. At the concentration of 10 μM, T3 and T4 depressed contractile response to 34.2 ± 5.9% and 18.9 ± 3.3% from precontraction level, respectively (one-way ANOVA, *p* < 0.05). Thus, T4 depressed contraction stronger compared to T3.

In tissues T4 can be converted into T3 by 5′-deiodination (Castro et al., 1985; Coppola et al., 2005; Arrojo E Drigo and Bianco, 2011). To prove that relaxation observed in the presence of T4 is not due to T4-derived T3 we used iopanoic acid (inhibitor of 5′-deiodinases, IOP, 100 μM). IOP did not change T4 effect (Figure 1C) meaning that T4 contributed substantially to the observed vasorelaxation.

Further we studied the effects of T3 and T4 on contractile responses to increasing concentrations of MX. This protocol provided more stable contractile responses of the sural arteries and allowed us to reduce the volume of vehicle added to the myograph chamber. Arteries were exposed to two consecutive CRRs to MX with TH added 20–30 min before the second CRR (for details see “Materials and Methods”). In these experiments, T3 and T4 were applied in the same concentration (10 μM). As shown in the Figures 2A,B, incubation with both forms of TH depressed the contractile responses to MX. This result is in accordance with T3- and T4-induced vasorelaxation observed in the previous protocol. To explore the molecular pathways behind T3- and T4-induced relaxation we used protocol with two consecutive CRRs to MX in further analysis.

### The Role of the Endothelium in the Vasorelaxation Induced by T3 and T4

To examine whether the suppression of contractile responses to MX induced by TH is endothelium-dependent, we removed the endothelium from the arteries and then studied the effects of TH on MX-induced contractions (Figures 2C,D). The effects of T3 and T4 were more pronounced in the middle region of the MX CRR: both hormones reduced MX-sensitivity of endothelium intact arteries (Table 1), while E_max_ was moderately reduced by T3 but not changed by T4 (Table 2). In endothelium-denuded preparations, T3 was no longer able to weaken MX-induced contractions (Figure 2C and Table 1). However, T4 was still able to reduce contraction to MX in endothelium-denuded arteries (Figure 2D and Table 1). Moreover, the magnitude of overall T4 effect on contraction estimated by AUC (% of respective control) was similar in endothelium-intact and endothelium-denuded segments (AUC decreased to 72.4 ± 8.2% and 75.0 ± 4.9%, respectively, *p* > 0.05).

**TABLE 1:** Comparison of EC_50_ values (concentration evoked half-maximal response) calculated for methoxamine concentration-response relationships shown in Figures 2–4, 6, 7.

Figure	Group 1		Group 2	
	Treatment	EC50, μ M	Treatment	EC50, μ M
2A	Vehicle (*n* = 8)	1.48 ± 0.30	T3 (*n* = 8)	2.6 ± 0.39*
2B	Vehicle (*n* = 7)	3.84 ± 0.83	T4 (*n* = 7)	16.29 ± 6.15*
2C	Vehicle (*n* = 7)	1.58 ± 0.44	T3 (*n* = 7)	1.07 ± 0.20
2D	Vehicle (*n* = 12)	1.44 ± 0.13	T4 (*n* = 10)	4.28 ± 0.76*
3A	Vehicle (*n* = 10)	1.71 ± 0.21	T4 (*n* = 10)	5.08 ± 1.20*
3B	L-NNA (*n* = 10)	1.34 ± 0.05	L-NNA + T4 (*n* = 10)	4.56 ± 1.51*
4A	Vehicle (*n* = 7)	0.96 ± 0.08	T4 (*n* = 7)	3.44 ± 0.53*
4B	Tetrac (*n* = 7)	1.82 ± 0.27	Tetrac + T4 (*n* = 7)	3.94 ± 1.11
6A	U0124 (*n* = 7)	1.20 ± 0.20	U0124 + T4 (*n* = 7)	3.95 ± 0.95*
6B	U0126 (*n* = 7)	1.56 ± 0.28	U0126 + T4 (*n* = 7)	3.45 ± 1.21
6C	Vehicle (*n* = 9)	1.80 ± 0.25	T4 (*n* = 9)	7.50 ± 1.86*
6D	Cpd22 (*n* = 9)	6.28 ± 1.89	Cpd22 + T4 (*n* = 9)	8.62 ± 2.32
7A	PP3 (*n* = 8)	1.53 ± 0.19	PP3 + T4 (*n* = 8)	5.30 ± 1.16*
7B	PP2 (*n* = 8)	7.70 ± 2.41	PP2 + T4 (*n* = 8)	22.8 ± 5.35*
7C	Vehicle (*n* = 7)	1.32 ± 0.15	T4 (*n* = 7)	3.33 ± 0.70*
7D	Y27632 (*n* = 7)	6.22 ± 1.12	Y27632 + T4 (*n* = 7)	13.6 ± 2.00*

*p < 0.05 compared to corresponding Group 1 (Unpaired Student’s *t*-test).

**TABLE 2:** Comparison of the maximum response (E_max_) calculated for methoxamine concentration-response relationships shown in Figures 2–4, 6, 7.

Figure	Group 1		Group 2	
	Treatment	E_max_, %	Treatment	E_max_, %
2A	Vehicle (*n* = 8)	95.8 ± 1.8	T3 (*n* = 8)	89.8 ± 1.6*
2B	Vehicle (*n* = 7)	95.8 ± 1.2	T4 (*n* = 7)	99.5 ± 5.7
2C	Vehicle (*n* = 7)	97.3 ± 0.6	T3 (*n* = 7)	95.2 ± 1.6
2D	Vehicle (*n* = 12)	98.2 ± 1.3	T4 (*n* = 10)	96.2 ± 1.6
3A	Vehicle (*n* = 10)	98.7 ± 0.8	T4 (*n* = 10)	90.9 ± 1.6*
3B	L-NNA (*n* = 10)	105.8 ± 0.9	L-NNA + T4 (*n* = 10)	107.5 ± 3.5
4A	Vehicle (*n* = 7)	99.7 ± 1.2	T4 (*n* = 7)	95.6 ± 2.8
4B	Tetrac (*n* = 7)	90.2 ± 1.4	Tetrac + T4 (*n* = 7)	90.6 ± 2.8
6A	U0124 (*n* = 7)	99.3 ± 1.6	U0124 + T4 (*n* = 7)	96.0 ± 1.1
6B	U0126 (*n* = 7)	92.4 ± 1.1	U0126 + T4 (*n* = 7)	92.0 ± 1.3
6C	Vehicle (*n* = 9)	96.2 ± 1.5	T4 (*n* = 9)	94.6 ± 3.1
6D	Cpd22 (*n* = 9)	89.4 ± 7.6	Cpd22 + T4 (*n* = 9)	93.9 ± 2.6
7A	PP3 (*n* = 8)	96.2 ± 1.2	PP3 + T4 (*n* = 8)	96.9 ± 3.8
7B	PP2 (*n* = 8)	78.4 ± 2.7	PP2 + T4 (*n* = 8)	80.9 ± 5.4*
7C	Vehicle (*n* = 7)	99.6 ± 1.1	T4 (*n* = 7)	94.6 ± 1.2
7D	Y27632 (*n* = 7)	79.3 ± 1.2	Y27632 + T4 (*n* = 7)	73.9 ± 1.2*

*p < 0.05 compared to corresponding Group 1 (Unpaired Student’s *t*-test).

These data indicate that T3 and T4 induce acute non-genomic vasorelaxation via different mechanisms, endothelium-dependent and endothelium-independent, respectively. Of note, expression of all three NOS isoforms may be observed in arterial smooth muscle cells (Carrillo-Sepulveda et al., 2010). However, NOS inhibitor N^ω^ -Nitro-L-arginine did not abolish the relaxatory effect of T4 (Figures 3A,B and Table 1). In further studies we focused on T4-induced relaxation, since, to the best of our knowledge, neither the receptor nor the key cascade participants of such effect have yet been identified.

### Integrin avb3 Participation in the Acute Effect of T4

In order to identify the smooth muscle cell receptor mediating T4-induced relaxation we used tetrac (tetraiodothyroacetic acid), a competitive inhibitor of integrin αvβ3-T4 interaction (Lin et al., 1999a; Bergh et al., 2005). Tetrac abolished T4-induced suppression of contractile responses to MX in endothelium-denuded arteries (Figures 4A,B and Table 1). Apparently, integrin αvβ3 mediates T4-induced non-genomic vasorelaxation in skeletal muscle arteries.

### The Effect of T4 on MLC2 Phosphorylation

**FIGURE 5:**
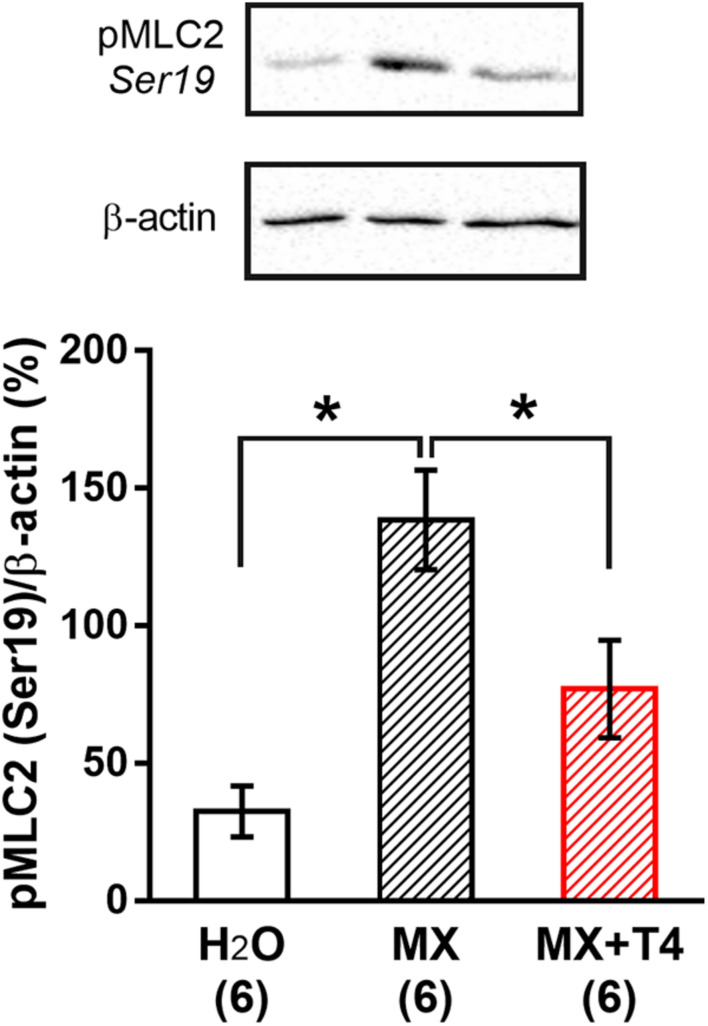
T4 weakens the methoxamine-induced increase in phospho-MLC2 (Ser19) content in sural arteries. The representative membrane parts are shown in the top. Data were normalized to β-actin level in the same sample and then the average value of two reference samples was taken as 100%. Number in parentheses represents the number of animals. *p < 0.05 (one-way ANOVA with Dunnett *post hoc* test).

Smooth muscle contraction is predominantly regulated by regulatory myosin light chain (MLC2) phosphorylation at Ser19 (Kamm and Stull, 2001; Somlyo and Somlyo, 2003; Mizuno et al., 2008). Therefore, studying signaling pathways involved in T4-induced vasorelaxation, we decided to investigate if the depressed contraction observed in the presence of T4 is associated with a change in the level of MLC2 phosphorylation at Ser19. MX (10 μM) increased the content of phospho-MLC2 (Ser19) compared to control arteries incubated with vehicle (H_2_O). The rise of phospho-MLC2 content in response to MX was less prominent in samples preincubated with T4 (10 μM) (Figure 5). These data suggest that T4-induced suppression of arterial contraction is realized, at least partially, by the thick filament regulatory pathway.

### The Impact of ERK1/2 and ILK Inhibitors on the T4-Induced Relaxation of Endothelium-Denuded Arteries

**FIGURE 6:**
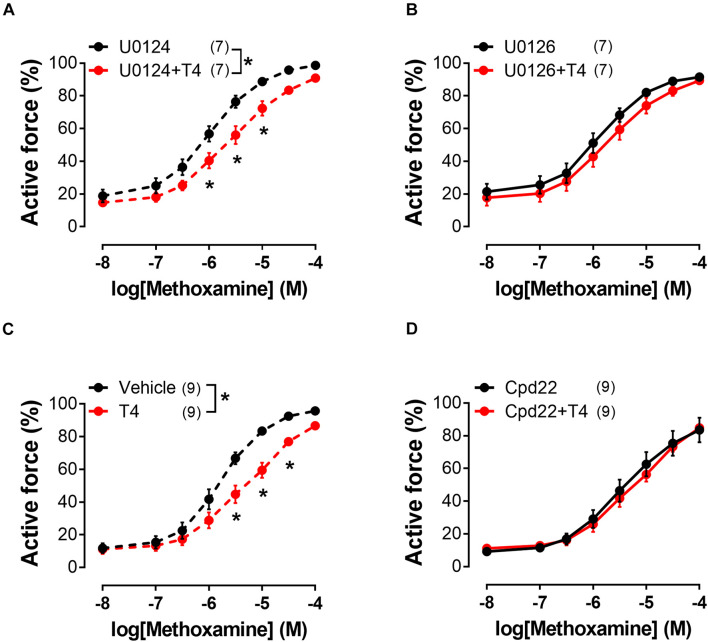
Inhibitor of ERK1/2 activation (U0126) and inhibitor of ILK (Cpd22) abolish the effects of T4 on contractile responses of endothelium-denuded sural arteries to methoxamine. Concentration-response relationships to methoxamine obtained after preincubation with T4 or vehicle in the presence of: U0124 **(A)** (inactive analogue of U0126, 10 μM), U0126 **(B)** (10 μM), DMSO **(C)** (vehicle for Cpd22), and Cpd22 **(D)** (10 μM). Number in parentheses represents the number of animals. *p < 0.05 compared to respective control group (Repeated Measures ANOVA with Tukey’s *post hoc* test).

To identify the signaling pathways which may transduce the T4-induced signal from integrin αvβ3 to the contractile apparatus of the cell and MLC2 in particular, we further performed an analysis using inhibitors of potentially involved kinases. One of the kinases that might participate in integrin signaling is ERK1/2 (Rucci et al., 2005). U0126 (10 μM), an inhibitor of MEK1/2 which are upstream to ERK1/2, abolished T4 effects on MX-induced contractile responses, while in the presence of its inactive analogue U0124 (10 μM) the effect of T4 was still present (Figures 6A,B and Table 1). Another kinase that may be involved in integrin αvβ3 signaling and MLC2 phosphorylation is integrin-linked kinase (ILK) (Deng et al., 2001). In the presence of ILK inhibitor compound22 (Cpd22, 10 μM) T4 was not able to reduce contractile responses to MX (Figures 6C,D and Table 1). The data obtained indicate that both ERK1/2 and ILK mediate the acute effect of T4.

### The Impact of Src- and Rho-Kinase Inhibitors on the T4-Induced Relaxation of Endothelium-Denuded Arteries

**FIGURE 7:**
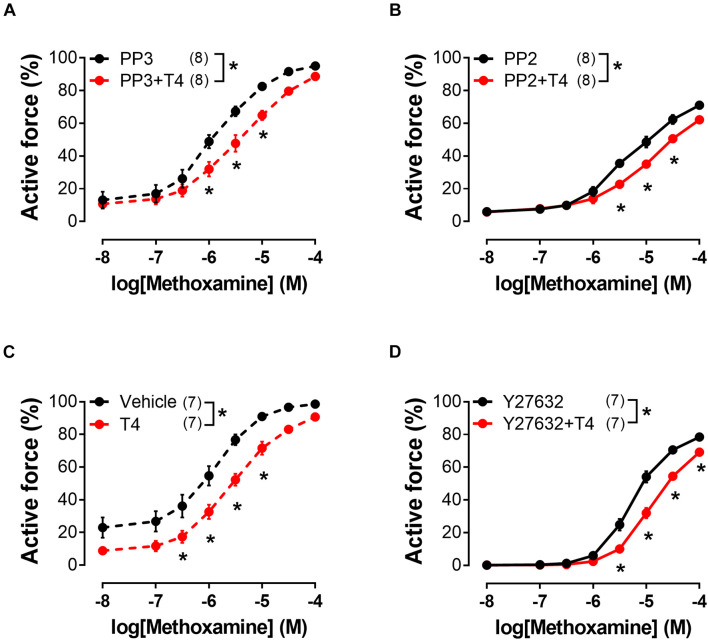
T4-induced suppression of contractile responses of endothelium-denuded sural arteries to methoxamine persists in the presence of Src-kinase (PP2) and Rho-kinase (Y27632) inhibitors. Concentration-response relationships to methoxamine obtained after preincubation with T4 or vehicle in the presence of PP3 **(A)** (inactive analogue of PP2, 10 μM), PP2 **(B)** (10 μM), H_2_O **(C)** (vehicle for Y27632), and Y27632 **(D)** (3 μM). Number in parentheses represents the number of animals. *p < 0.05 compared to respective control group (Repeated Measures ANOVA with Tukey’s *post hoc* test).

Src-kinase may be also activated by integrin αvβ3 (Qiu et al., 2014; Flamini et al., 2017). Activation of Src-kinase may lead to the Rho-kinase activation (Samarakoon et al., 2011), which is an important regulator of myosin light chain phosphatase (MLCP) activity (Qiao et al., 2014). In our study, T4 suppressed the MX-induced contraction in the presence of Src-kinase inhibitor PP2 (10 μM), as well as in the presence of its inactive analogue PP3 (10 μM) (Figures 7A,B and Table 1). Further, in the presence of Rho-kinase inhibitor Y27632 (3 μM) T4 was still able to suppress the MX-induced contraction (Figures 7C,D and Table 1). T4 reduced AUC (% of respective control) similarly in arteries treated with PP2 and PP3 (10 μM) (to 76.2 ± 4.8 and 77.7 ± 5.8%, respectively, *p* > 0.05). Accordingly, in arteries treated with Y27632 or vehicle AUC was also similarly reduced by T4 to 68.4 ± 4.4% and 70.3 ± 5.2%, respectively (*p* > 0.05). These data demonstrate that Src- or Rho-kinase activation is not essential for T4-induced suppression of contraction.

## Discussion

In this study, we have demonstrated that both T3 and T4 can induce acute, i.e., non-genomic relaxation of the rat skeletal muscle arteries, acting via essentially different mechanisms. The effect of T3 was endothelium-dependent, while the effect of T4 was endothelium-independent and more pronounced in this type of arteries. For the first time, we have studied the mechanisms of acute T4-induced vasorelaxation, showing that the relaxatory effect of T4 is mediated by integrin αvβ3 and is associated with the suppression of its intracellular signaling with participation of ERK1/2 and ILK kinases. We also showed that T4 causes a decrease in the phospho-MLC2 (Ser19) content, meaning that the T4-induced vasorelaxation is realized, at least partially, by the thick filament regulatory pathway.

### T3 and T4 Induce Vasorelaxation of Skeletal Muscle Arteries via Different Mechanisms

We have shown that both T3 and T4 can induce relaxation of the rat skeletal muscle arteries within a few minutes, which allowed us to consider the observed relaxation as non-genomic, since the regulation of transcription is a more time-consuming process. The effect of T4 was more pronounced than that of T3, similar to the results obtained by the Zwaveling et al. (1997) in the mesenteric arteries. Along with that, in coronary arteries two hormones showed similar potency (Yoneda et al., 1998). Finally, in the study by Park et al. (1997), T3 caused more pronounced relaxation of skeletal muscle arterioles than T4 (Park et al., 1997). This inconsistency of the results suggests that different receptors and signaling pathways could be involved in the TH-induced relaxation depending on the vascular region or certain experimental conditions.

Of note, the concentrations at which T4 or T3 are able to induce relaxation *in vitro* usually exceed their blood concentrations (Zwaveling et al., 1997; Carrillo-Sepulveda et al., 2010; Cai et al., 2015; Lozano-Cuenca et al., 2016; Gaynullina et al., 2018a). However, acute TH-induced vasorelaxation cannot be regarded as an experimental phenomenon only. In experiments on more distal branches of the vascular bed, TH induce acute relaxation at concentrations near the physiological range: T3 can cause dilation of arterioles at a concentration of several nM, and T4—of 150 nM (Park et al., 1997; Colantuoni et al., 2005). Along with that, larger arteries are commonly used in mechanistic Western blotting experiments, to obtain sufficient tissue volume for analysis. Importantly, vessel sensitivity to TH is higher under *in vivo* compared to *in vitro* conditions (Yoneda et al., 1998; Colantuoni et al., 2005; Napoli et al., 2007), which makes results of studies utilizing supraphysiological TH concentrations more relevant.

We have discovered that T3 and T4 induce vasorelaxation through different mechanisms. The T3-induced effect was endothelium-dependent, similar to observed in the aorta (Lozano-Cuenca et al., 2016) and skeletal muscle arterioles (Park et al., 1997). The acute action of T3 in endothelial cells is mediated by the cytoplasmic TRα1 receptor and PI3K/Akt signaling cascade with a subsequent eNOS activation and NO production (Colantuoni et al., 2005; Hiroi et al., 2006; Aoki et al., 2015; Lozano-Cuenca et al., 2016; Geist et al., 2021). Presumably, similar mechanisms are involved in the observed endothelium-dependent T3-induced relaxation of rat sural arteries.

In contrast to T3, relaxatory effect of T4 on skeletal muscle arteries was manifested regardless of the presence of endothelium in the arteries and, therefore, was independent of endothelium-derived NO, as opposed to T4 effect in rat mesenteric arteries (Zwaveling et al., 1997). Of note, eNOS expression level is two-fold higher in rat mesenteric arteries compared to sural arteries (Sofronova et al., 2016), suggesting different ability of endothelium to produce NO in these vascular beds. Moreover, our experiments with L-NNA showed that NO derived from either vascular endothelium or smooth muscle cells is unlikely involved in the effect of T4 (Figure 3).

To the best of our knowledge, the mechanisms of endothelium-independent T4-induced acute vasorelaxation have not been previously studied. Therefore, we addressed them in following experiments.

### T4-Induced Vasorelaxatory Effect Is Mediated by Integrin αvβ3, ERK1/2 and ILK, Not by Src- or Rho-Kinases

The relaxatory effect of T4 was not observed in the presence of its deaminated derivative tetrac, which interferes with the T4 binding to αvβ3 integrin (Bergh et al., 2005). It is known that, in blood vessels, integrin αvβ3 functions as a receptor for TH initiating angiogenesis (Liu et al., 2014). Importantly, the role of this receptor in non-genomic vasorelaxation was first discovered in our study. Integrin αvβ3 is expressed in both endothelial and smooth muscle vascular cells (Belmadani et al., 2008; Daeichin et al., 2016; Jenkins et al., 2019). Since T4-induced vasorelaxation is endothelium-independent, T4 effect on rat sural artery is initiated by the binding of the hormone to the αvβ3 integrin of smooth muscle cells. We assume that T4 induces vasorelaxation by binding to S2 site of αvβ3 integrin because S2 has higher affinity to T4 compared to T3 (Lin et al., 2009).

The degree of smooth muscle cell contraction is associated with the level of phosphorylation of MLC2 (Ser19) (Somlyo and Somlyo, 2003; Webb, 2003). We have shown that T4 weakens the rise in phospho-MLC2 (Ser19) content in response to the α_1_-adrenoceptor agonist MX, which is consistent with the relaxing effect of T4 on arteries observed in myograph experiments. These data suggest that signaling pathways mediating acute effect of T4: (i) must be functionally linked to integrin αvβ3 and (ii) must directly or indirectly affect the level of phospho-MLC2 (Ser19). A wide range of kinases match these criteria, including ERK1/2 (Rucci et al., 2005; Zeller et al., 2013; Barbakadze et al., 2014), ILK (Wilson et al., 2005; Dwivedi et al., 2008), Src-kinase (Knock et al., 2008), Rho-kinase (Knock et al., 2008; Chen et al., 2015), PI3K (Su et al., 2004), and Akt (Barbakadze et al., 2014).

We performed an inhibitory analysis of signaling pathways that may be associated with integrin αvβ3 and changes in the degree of phosphorylation of MLC2 (Ser19) and found that ERK1/2 and ILK kinases are involved in T4-induced vasorelaxation. It should be noted that our data do not allow us to directly establish the order of the ERK1/2 and ILK kinases in this cascade. However, we assume that ILK is a downstream kinase, since it is able to affect the phosphorylation of MLC2 directly or indirectly by inhibiting the MLCP activity (Deng et al., 2001, 2002; Murányi et al., 2002; Wilson et al., 2005). This order of kinases in cascade is also supported by the other studies that revealed the ability of ERK1/2 to activate ILK in visceral smooth muscles (Harnett et al., 2005; Ihara et al., 2007). Thus, the signaling pathway behind T4-induced vasorelaxation includes integrin αvβ3, ERK1/2 and ILK.

Integrin signaling can also be associated with activation of Src-kinase (Mao et al., 2012). First, Src-kinase can activate Rho-kinase (Knock et al., 2008), which, in turn, suppresses the activity of MLCP, increasing the phospho-MLC2 content (Li et al., 2014; Liu and Khalil, 2018). However, the T4 effect persisted in the presence of Src- and Rho-kinase inhibitors, which suggests that they do not mediate T4-induced suppression of MX contraction. Second, Src-kinase is capable of activating kinase Akt (Li et al., 2014; Liu and Khalil, 2018), while the Src/Akt pathway has been shown to be involved in the non-genomic effects of thyroid hormones in cells of non-vascular tissues (Lin et al., 2009). However, in rat sural artery T4 did not affect the phosphorylation level of Akt at Ser473 (Supplementary Figure 1). Thus, we assume that Src/Akt pathway does not mediate the acute effect of T4 in skeletal muscle arteries.

### T4-Induced Vasorelaxation Is Associated With Suppression of Extracellular Matrix Signaling in Smooth Muscle Cells

**FIGURE 8:**
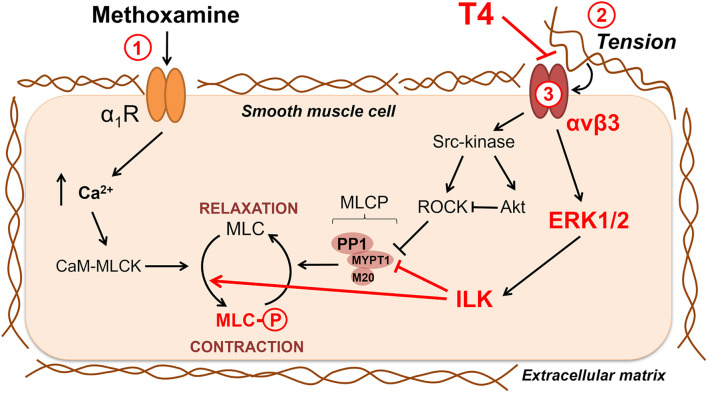
Potential signaling pathways involved in non-genomic relaxation of rat skeletal muscle artery in response to thyroxine. T4 induces endothelium-independent vasorelaxation due to suppression of extracellular matrix signaling, in detail: 1—contraction in response to α_1_-adrenoceptor agonist methoxamine increases arterial wall tension; 2—the tension activates integrin αvβ3 and downstream procontractile ERK1/2 signaling pathway which includes ILK and its targets MLCP and MLC; 3—T4 interacts with integrin αvβ3 at the site overlaying the site recognizing the extracellular matrix and, thereby, weakens the integrin signaling and methoxamine-induced contraction. Key participants of T4-induced vasorelaxation discovered in our study are shown in red. Src-kinase, ROCK and Akt also may participate in integrin-dependent signaling, but our data do not support their role in T4-induced vasorelaxation in skeletal muscle arteries. Sharp arrows show activation and blunt arrows—inhibition of the targets. α_1_R, α_1_-adrenoceptor; CaM, calmodulin; ILK, integrin-linked kinase; MLC, myosin light chain 2; MLCK, myosin light chain kinase; MLCP, myosin light chain phosphatase; MYPT1, PP1 and M20 are subunits of MLCP; ROCK, Rho-kinase.

We found that interaction of T4 with integrin αvβ3 induces vasorelaxation with participation of kinases ERK1/2 and ILK. This observation would seem to contradict the literature data, according to which the activation of ERK1/2 or ILK potentiates contraction of smooth muscle cells (D’Angelo and Adam, 2002; Ihara et al., 2007; Gaynullina et al., 2021). According to our hypothesis, T4 induces vasorelaxation by decreasing the activity of these kinases through attenuation of signals transmitted by integrin αvβ3 from the extracellular matrix (Figure 8). When smooth muscle cells contract in response to the α_1_-adrenoceptor agonist, this increases the tension of the artery wall (Takamizawa et al., 1992) and, therefore, changes the interaction between smooth muscle cells and extracellular matrix proteins (Hill and Meininger, 2012). Integrin αvβ3, as a part of the cell mechanotransduction system, is activated by tension in the extracellular matrix and triggers outside-in signaling cascades (Zeller et al., 2013; Chen et al., 2017). For instance, in fibroblast cultures, mechanical stretch leads to an integrin-dependent increase in the phospho-ERK1/2 content (Zeller et al., 2013). In smooth muscle cells, the integrin-mediated interaction of cells with the substrate increases phospho-MLC2 content (Polte et al., 2004). Since T4 binding site on the αvβ3 integrin partially overlaps with the binding site for extracellular matrix proteins (Cody et al., 2007), binding of T4 would weaken the interaction of integrin with the matrix and, therefore, reduce the intensity of the signal transmitted by it into the cell.

### Future Perspectives

The novel mechanism of T4-dependent control of mechanotransduction in vascular wall was identified here mostly at functional level. The reported mechanism expands the existing knowledge of the thyroid regulation of arterial tone, and in future it should be addressed at the molecular level. Future perspectives of such molecular study include (i) confirmation that ERK1/2 is upstream ILK in signaling pathway; (ii) evaluation of phospho-ERK1/2 contents in methoxamine-contracted arteries in presence of vehicle or T4; (iii) exploration of the mechanisms of procontractile action of ILK. Other potential targets of ILK include MYPT1 (phosphorylation at Thr709, Thr695, Thr495) (Murányi et al., 2002), MLCP inhibitors CPI-17 and PHI-1 (phosphorylation at Thr38 and Thr57, respectively) (Deng et al., 2002) and MLC2 (phosphorylation at Thr18) (Wilson et al., 2005). Of note, the Ca^2+^-independent myosin diphosphorylation by ILK could contribute to the stable contraction of the artery (Sutherland and Walsh, 2012).

## Conclusion

In this study, we report a novel mechanism of T4-induced acute non-genomic relaxation of skeletal muscle arteries. Our new data together with literature data are summarized in Figure 8. Our main idea is that T4 induces endothelium-independent vasorelaxation by suppressing the integrin signaling in smooth muscle cells. When a smooth muscle cell contracts, the extracellular matrix is stretched, which further enhances the contraction due to the activation of the αvβ3 integrin/ERK1/2/ILK cascade and phosphorylation of MLC2 (Ser19). T4 suppresses the binding of integrin to a stressed matrix and, therefore, weakens outside-in signal from αvβ3 integrin, which leads to relaxation of smooth muscle cell.

Importantly, skeletal muscle blood flow is up to 20% of cardiac output at rest and even more during exercise (Laughlin et al., 2012). Therefore, the results of our study point to a possible mechanism of decreased peripheral vascular resistance in hyperthyroidism. The other iodothyronines such as diiodothyronine or iodothyronamine should also be investigated as potential integrin αvβ3 ligands and vasoactive agents. The acute vasorelaxatory effects of T4 should be considered when prescribing hormone replacement therapy for people with thyroid axis disorders.

## Data Availability Statement

The original contributions presented in the study are included in the article/Supplementary Material, further inquiries can be directed to the corresponding author.

## Ethics Statement

The animal study was reviewed and approved by Biomedical Ethics Committee of the Russian Federation State Research Center Institute for Biomedical Problems, Russian Academy of Sciences.

## Author Contributions

ES, DG, and OT conceived and designed the study and analyzed the data. ES performed all myography experiments and drafted the manuscript. ES and DG performed Western blotting experiments. All authors contributed to the final writing and approved the version to be submitted.

## Conflict of Interest

The authors declare that the research was conducted in the absence of any commercial or financial relationships that could be construed as a potential conflict of interest.

## Publisher’s Note

All claims expressed in this article are solely those of the authors and do not necessarily represent those of their affiliated organizations, or those of the publisher, the editors and the reviewers. Any product that may be evaluated in this article, or claim that may be made by its manufacturer, is not guaranteed or endorsed by the publisher.
