# Supplementary material for: Thyroxine Induces Acute Relaxation of Rat Skeletal Muscle Arteries via Integrin αvβ3, ERK1/2 and Integrin-Linked Kinase
Source: Front Physiol. 2021 Sep 14;12:726354. doi: 10.3389/fphys.2021.726354 (PMC8477044; doi:10.3389/fphys.2021.726354)

Original unprocessed images of Western blot membranes used in the article

Samples used for analysis

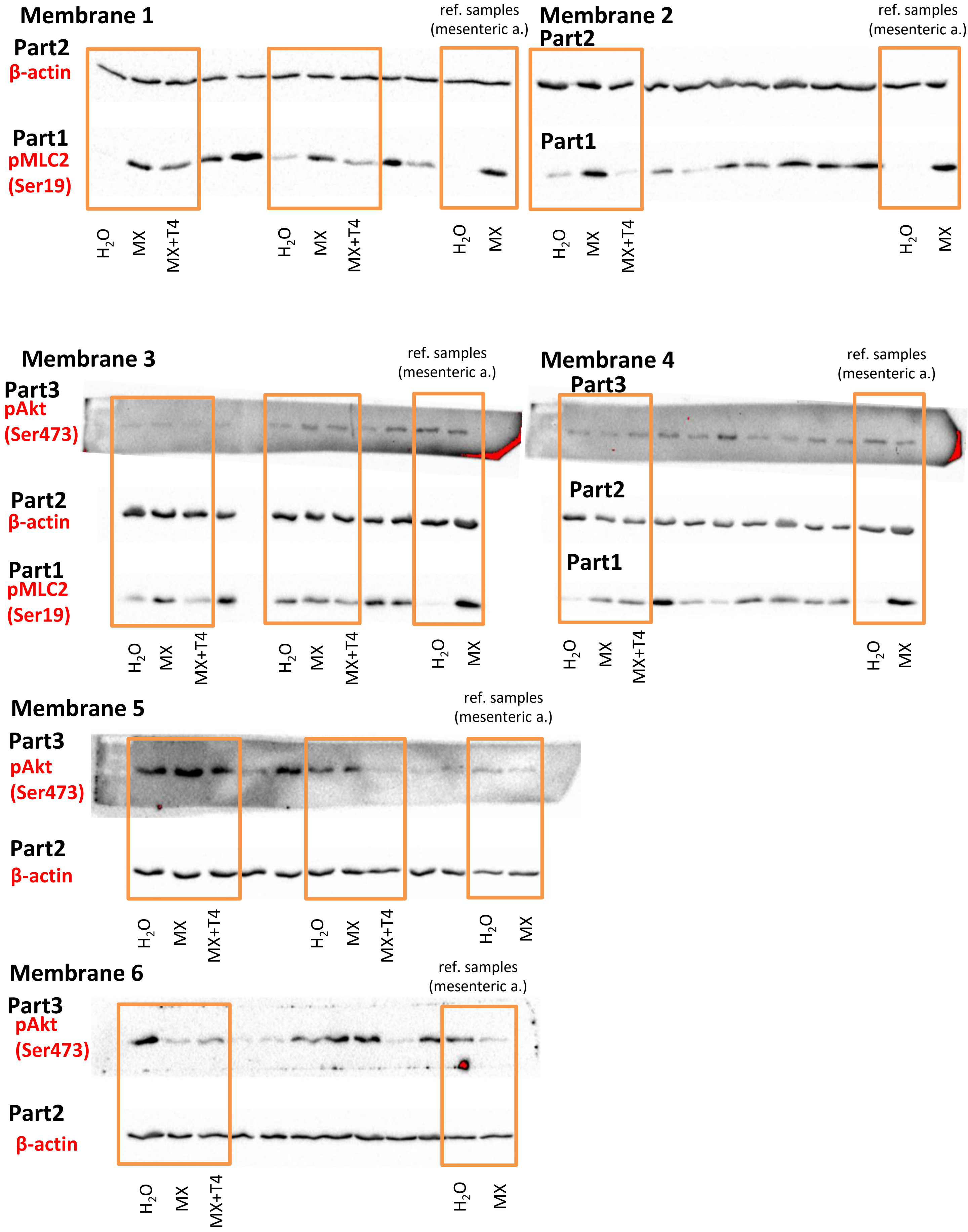

Supplement: Supplementary file 1 [file Data_Sheet_1.PDF]
