# Supplementary figures and images for: Thyroxine Induces Acute Relaxation of Rat Skeletal Muscle Arteries via Integrin αvβ3, ERK1/2 and Integrin-Linked Kinase
Source: Front Physiol. 2021 Sep 14;12:726354. doi: 10.3389/fphys.2021.726354 (PMC8477044; doi:10.3389/fphys.2021.726354)

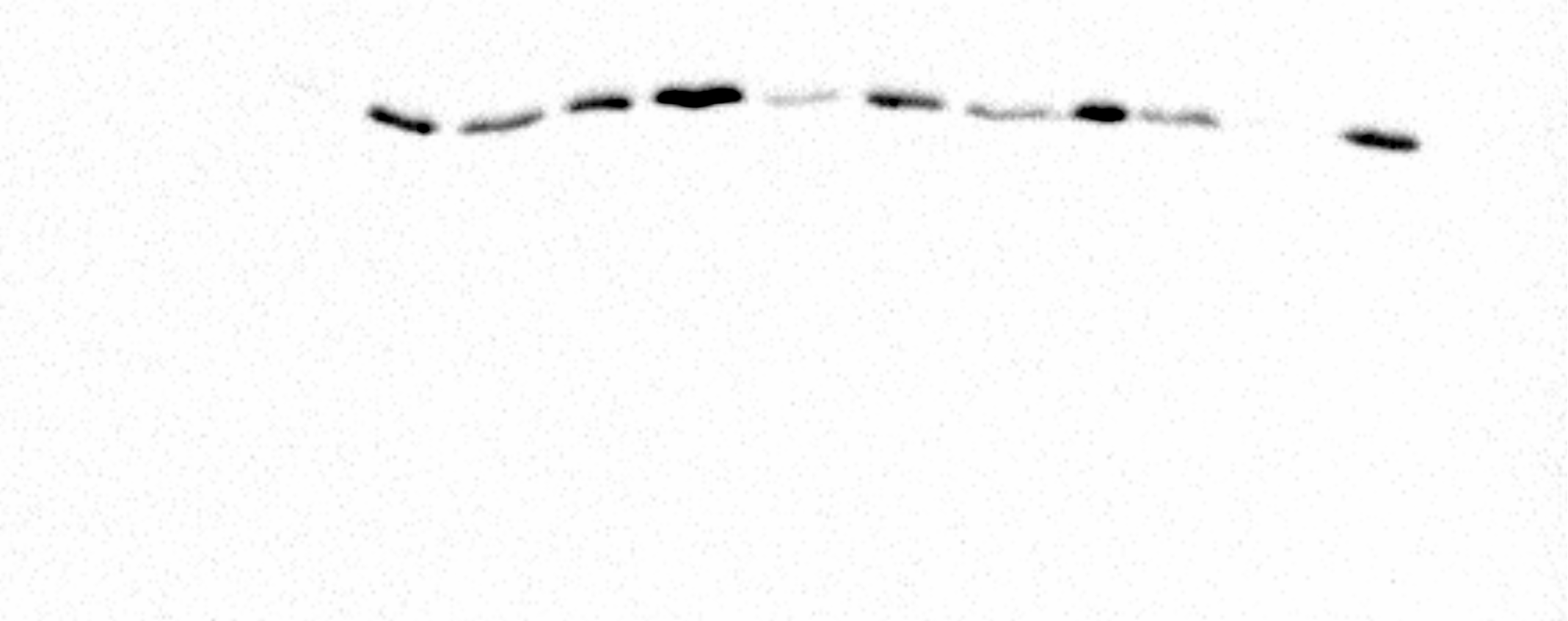

Supplement: Supplementary file 3 [file Data_Sheet_3.ZIP › Selivanova original blots single files/Membrane1-part1 (pMLC2-Ser19).tif]

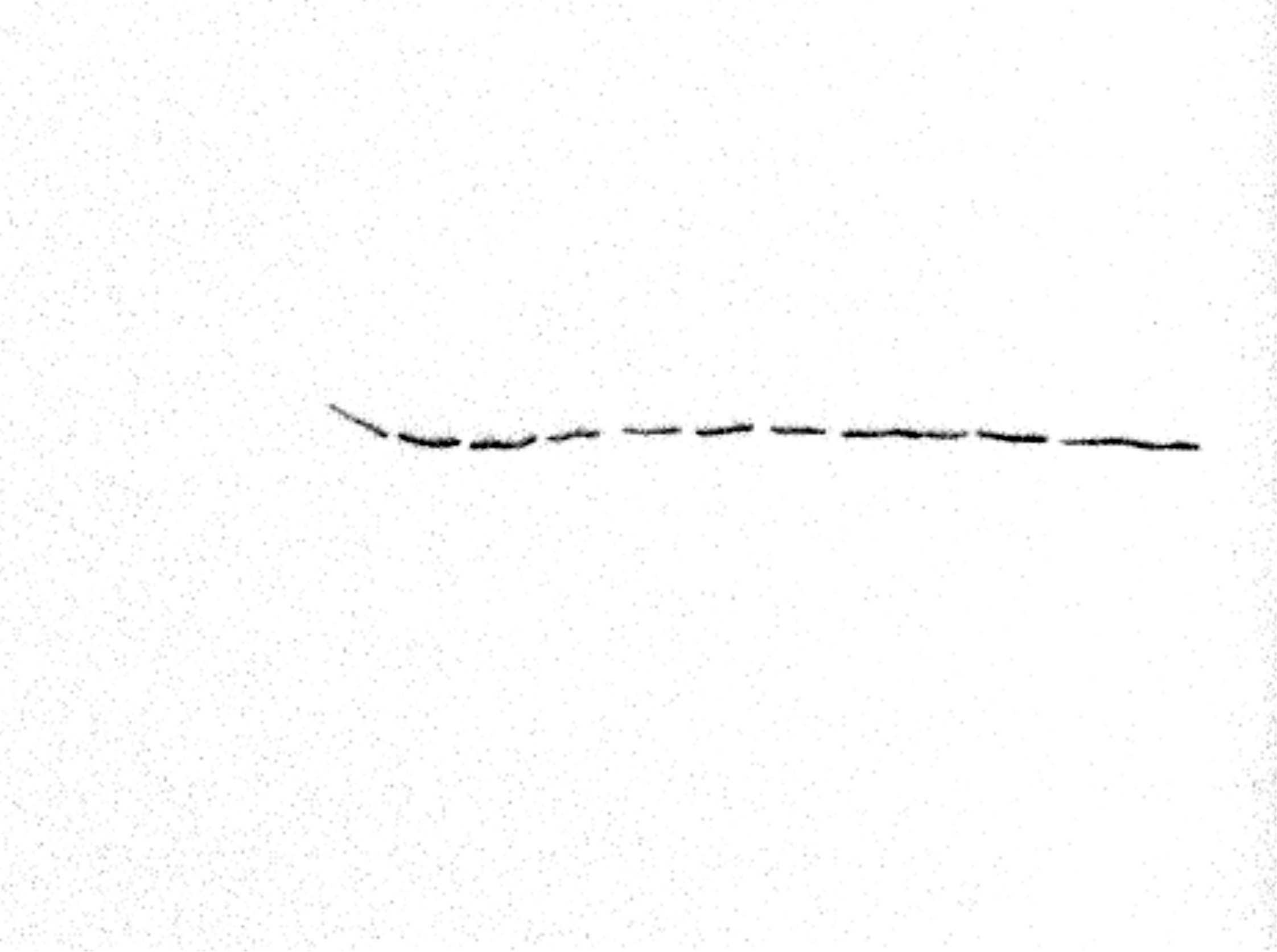

Supplement: Supplementary file 3 [file Data_Sheet_3.ZIP › Selivanova original blots single files/Membrane1-part2 (b-actin).tif]

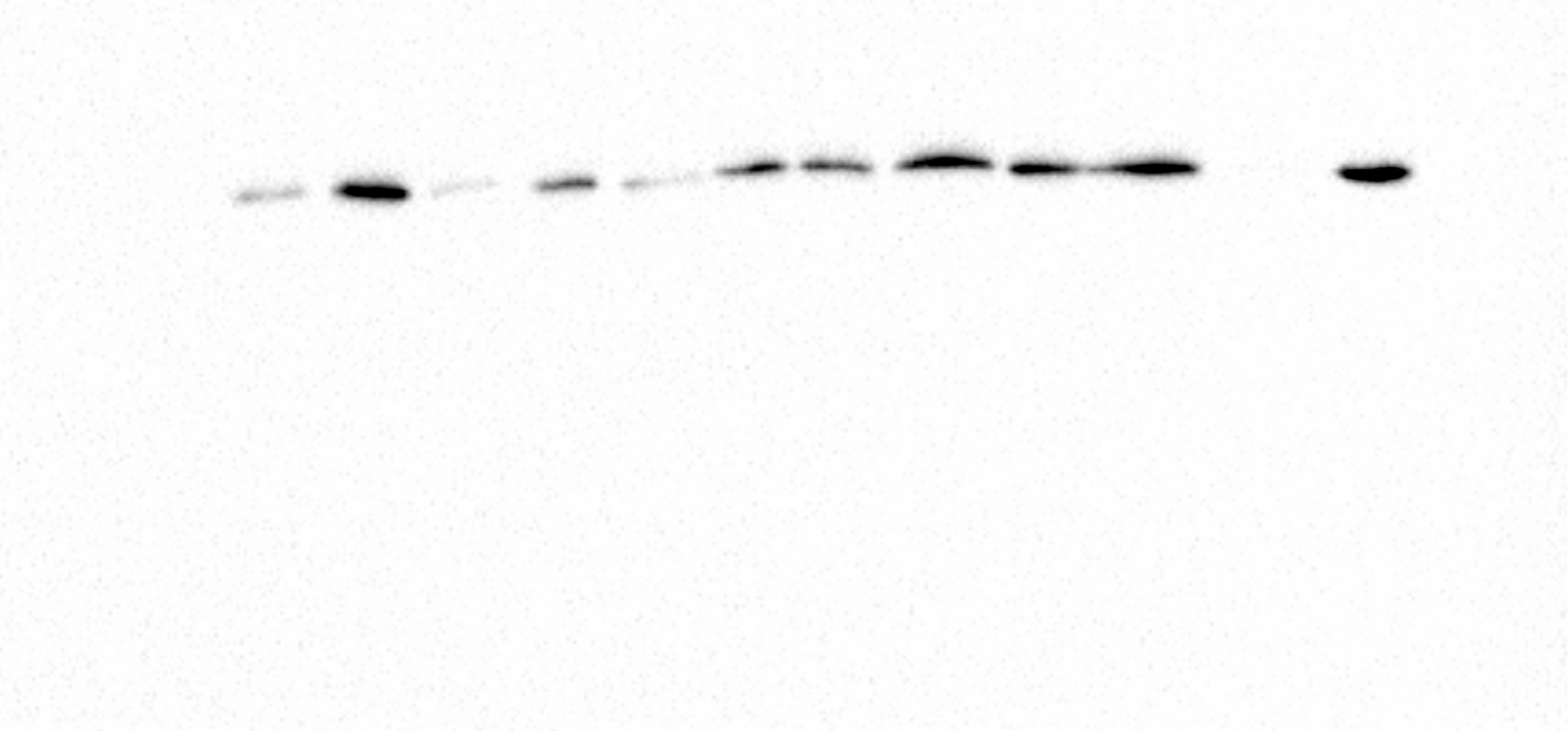

Supplement: Supplementary file 3 [file Data_Sheet_3.ZIP › Selivanova original blots single files/Membrane2-part1 (pMLC2-Ser19).tif]

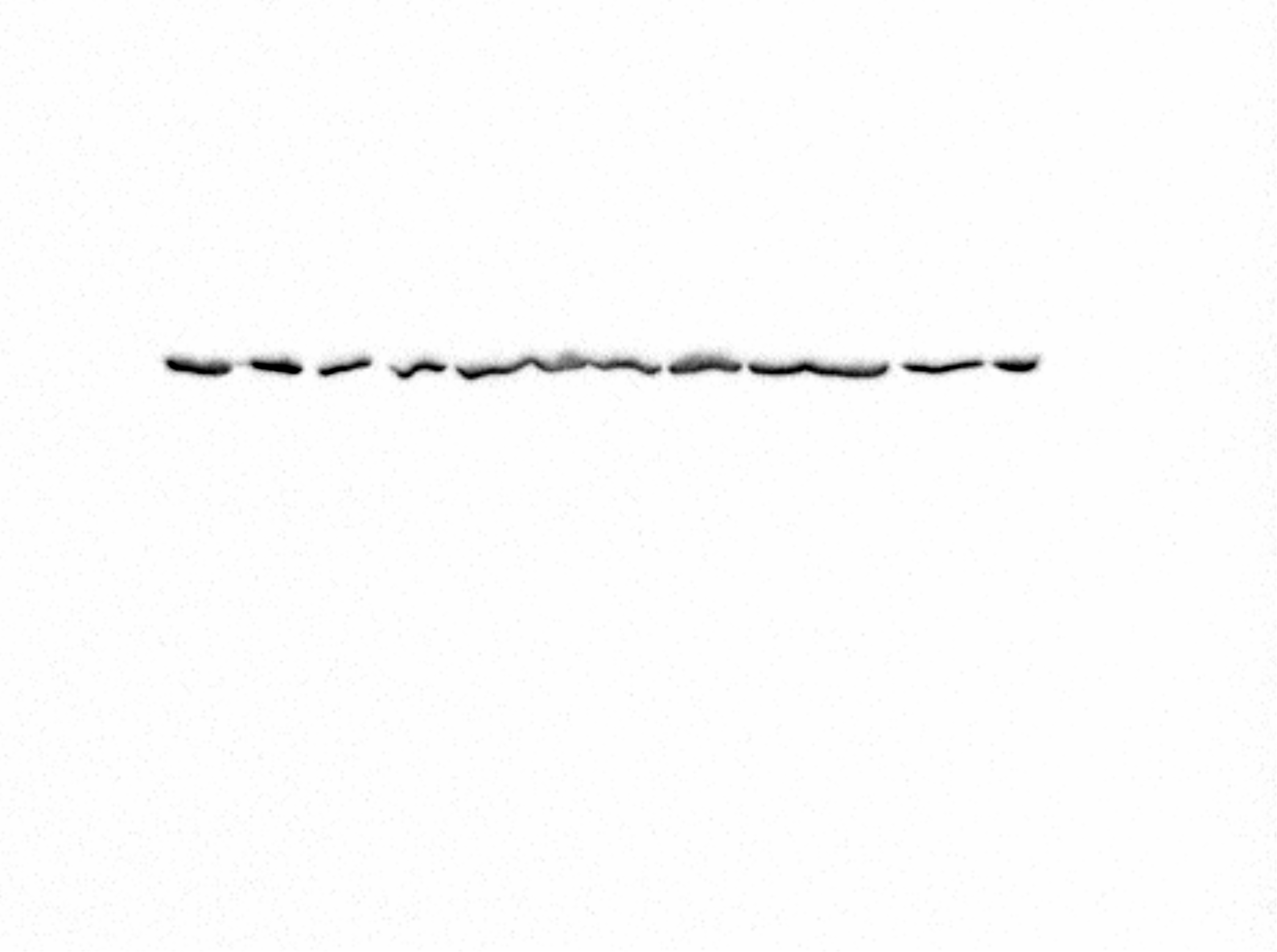

Supplement: Supplementary file 3 [file Data_Sheet_3.ZIP › Selivanova original blots single files/Membrane2-part2 (b-actin).tif]

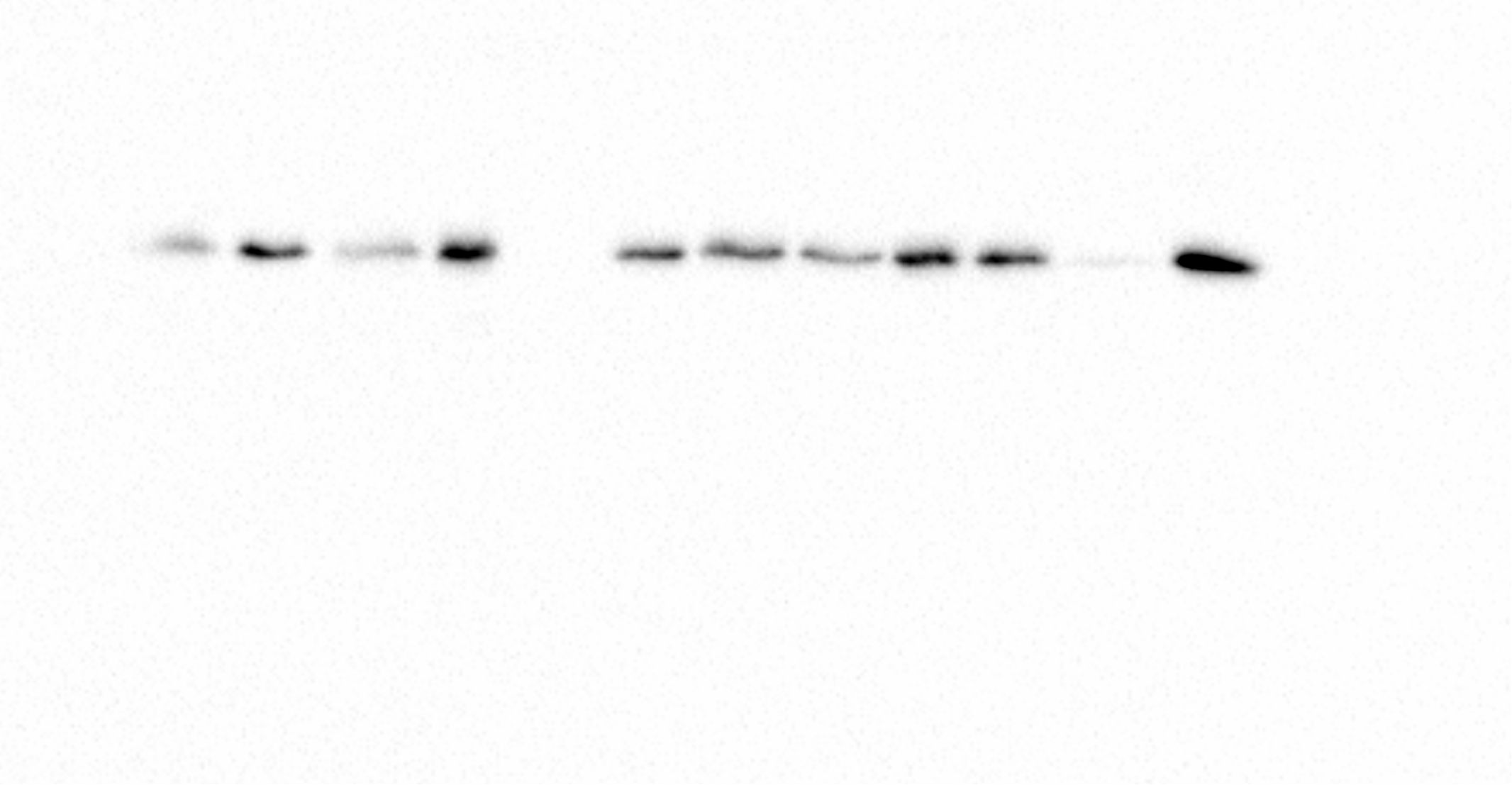

Supplement: Supplementary file 3 [file Data_Sheet_3.ZIP › Selivanova original blots single files/Membrane3-part1 (pMLC2-Ser19).tif]

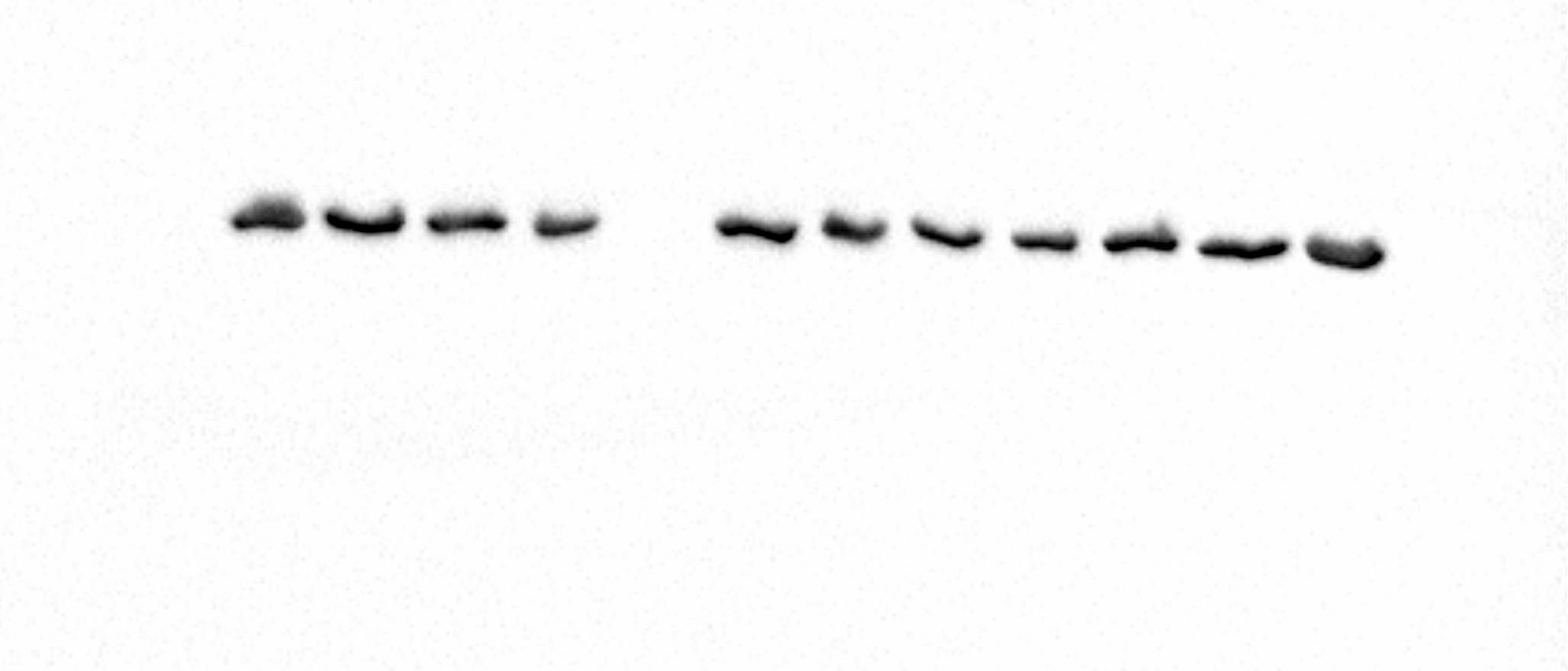

Supplement: Supplementary file 3 [file Data_Sheet_3.ZIP › Selivanova original blots single files/Membrane3-part2 (b-actin).tif]

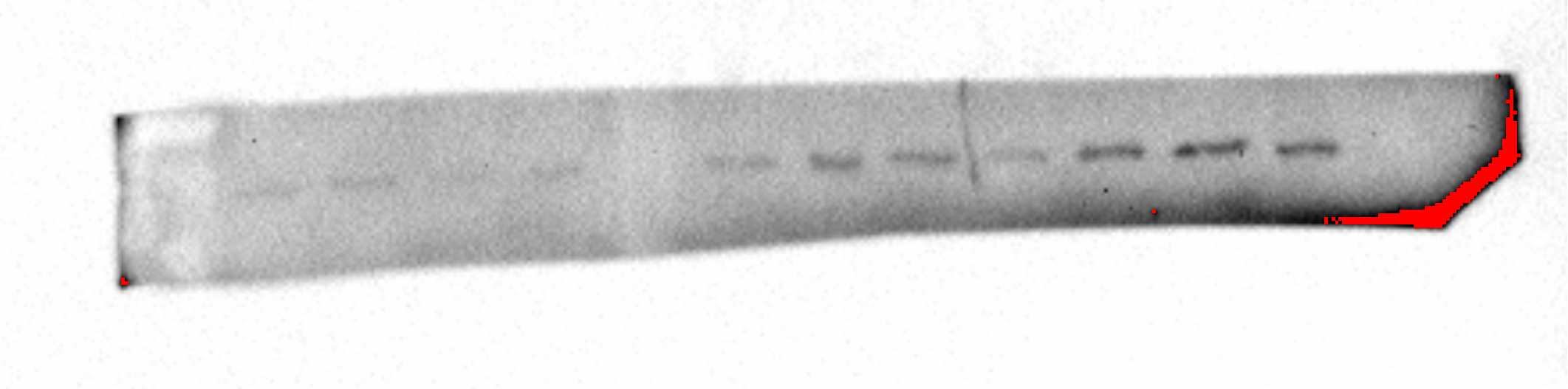

Supplement: Supplementary file 3 [file Data_Sheet_3.ZIP › Selivanova original blots single files/Membrane3-part3 (pAkt-Ser473).tif]

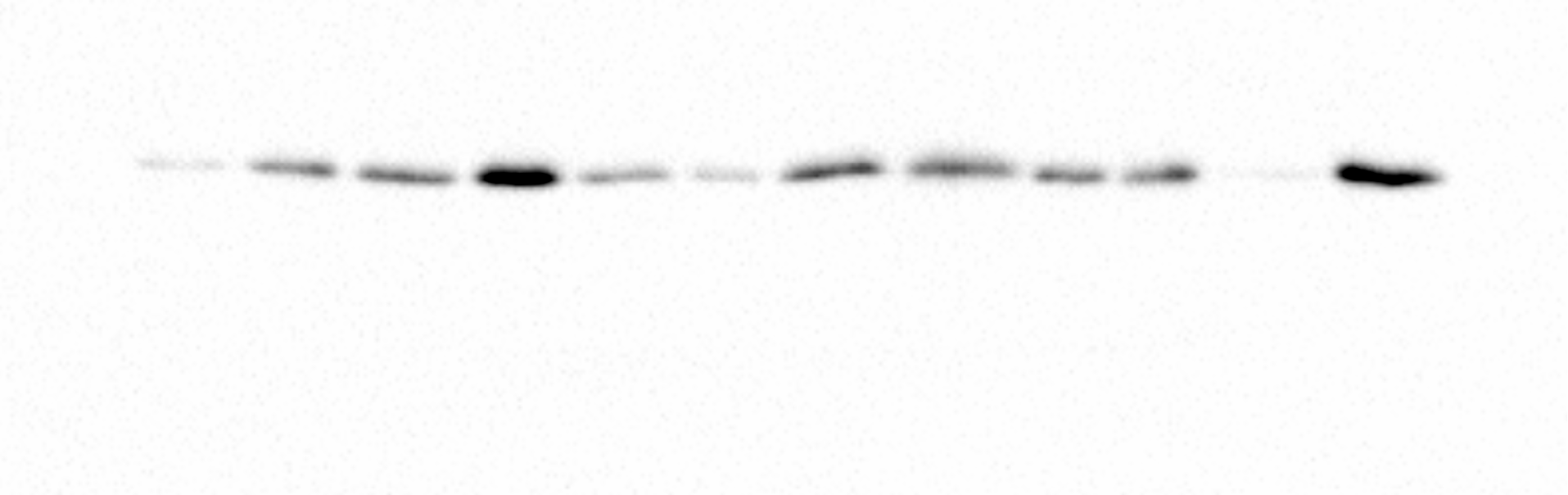

Supplement: Supplementary file 3 [file Data_Sheet_3.ZIP › Selivanova original blots single files/Membrane4-part1 (pMLC2-Ser19).tif]

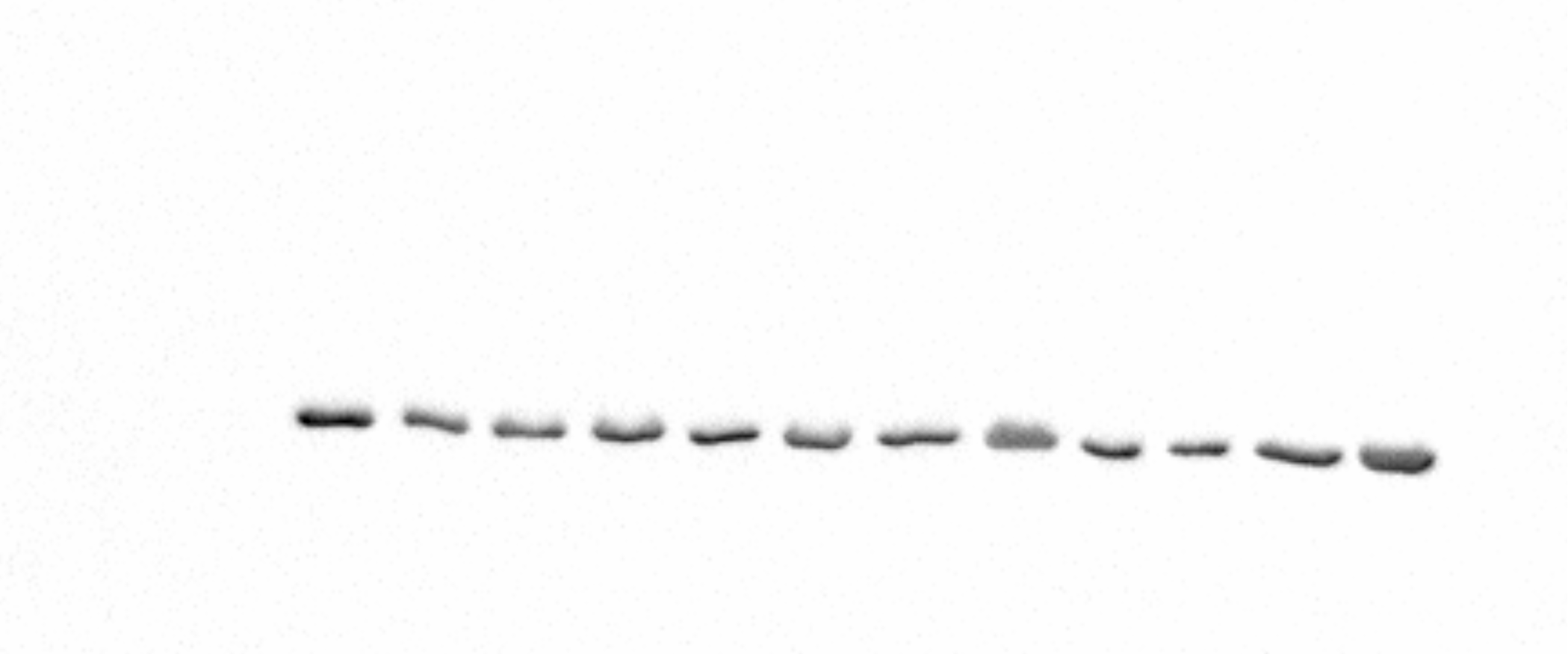

Supplement: Supplementary file 3 [file Data_Sheet_3.ZIP › Selivanova original blots single files/Membrane4-part2 (b-actin).tif]

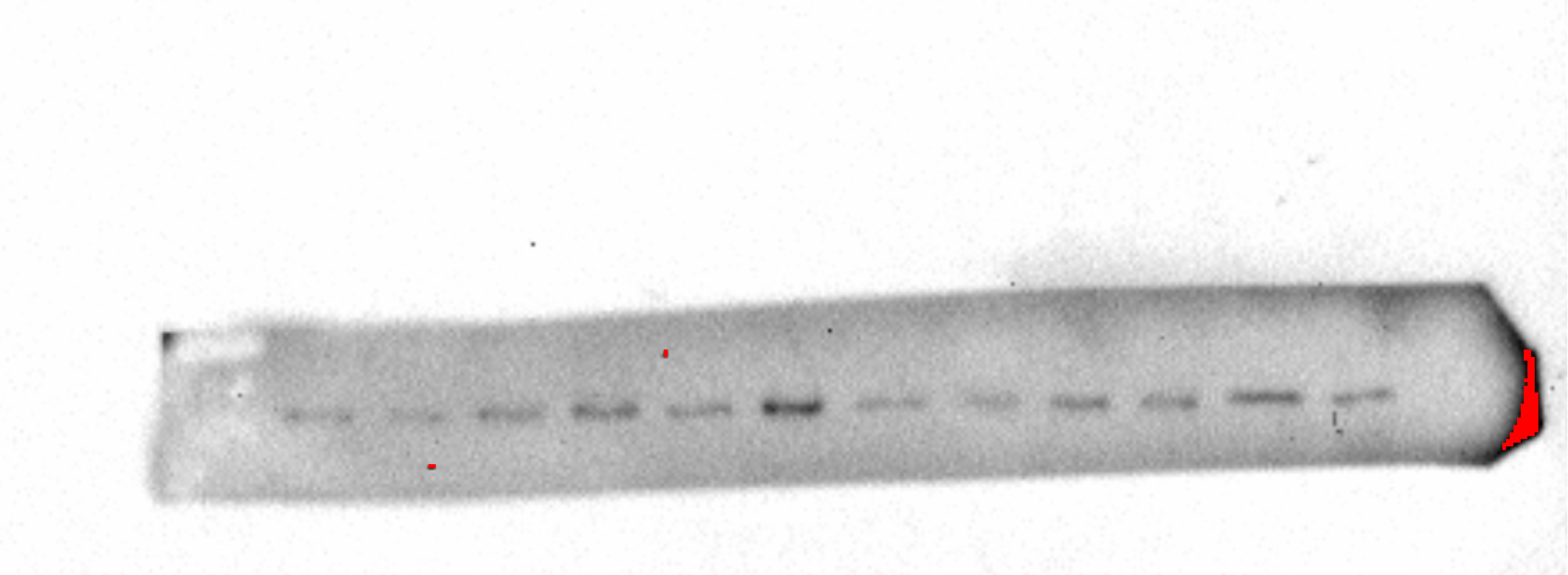

Supplement: Supplementary file 3 [file Data_Sheet_3.ZIP › Selivanova original blots single files/Membrane4-part3 (pAkt-Ser473).tif]

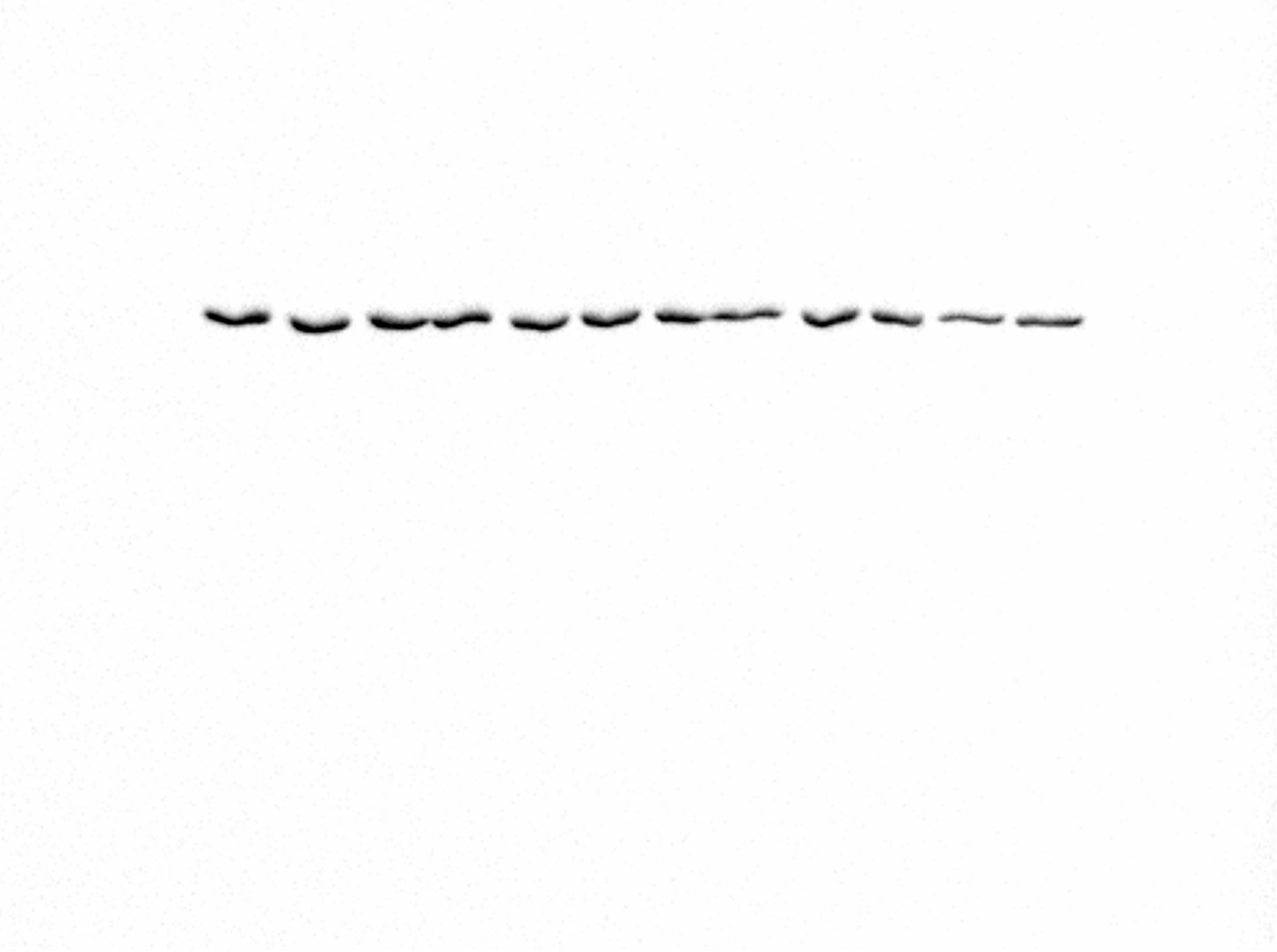

Supplement: Supplementary file 3 [file Data_Sheet_3.ZIP › Selivanova original blots single files/Membrane5-part2 (b-actin).tif]

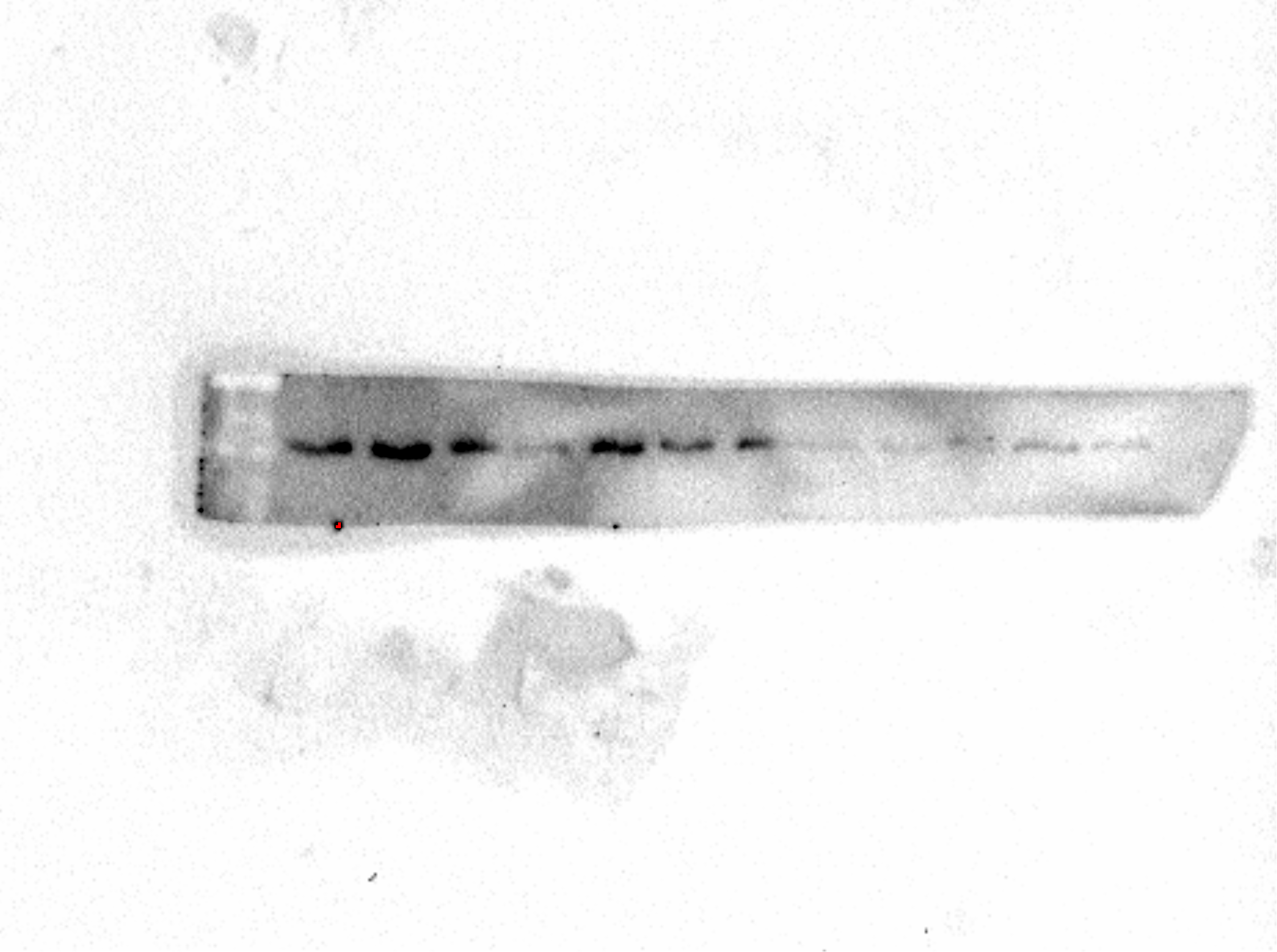

Supplement: Supplementary file 3 [file Data_Sheet_3.ZIP › Selivanova original blots single files/Membrane5-part3 (pAkt-Ser473).tif]

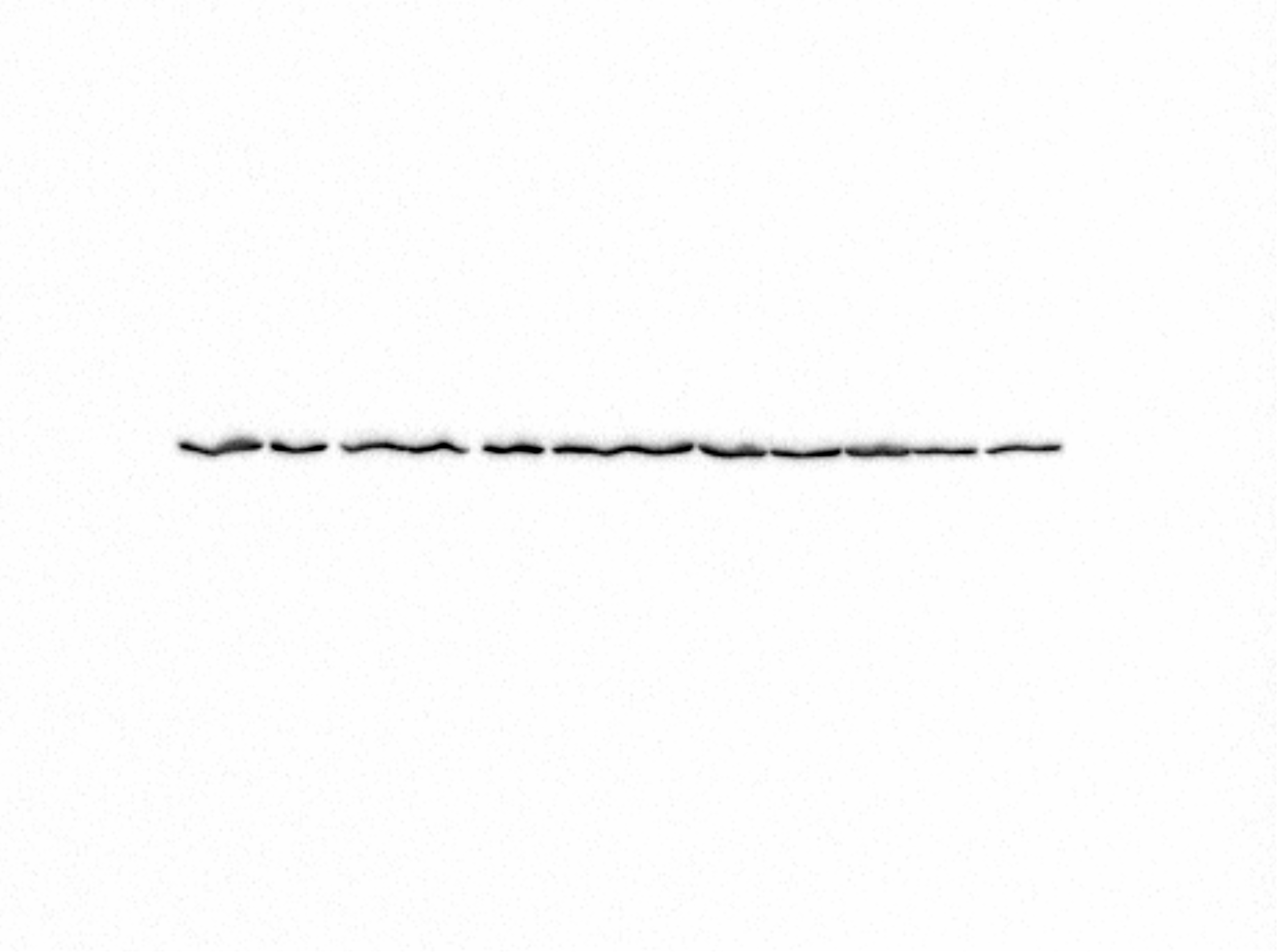

Supplement: Supplementary file 3 [file Data_Sheet_3.ZIP › Selivanova original blots single files/Membrane6-part2 (b-actin).tif]

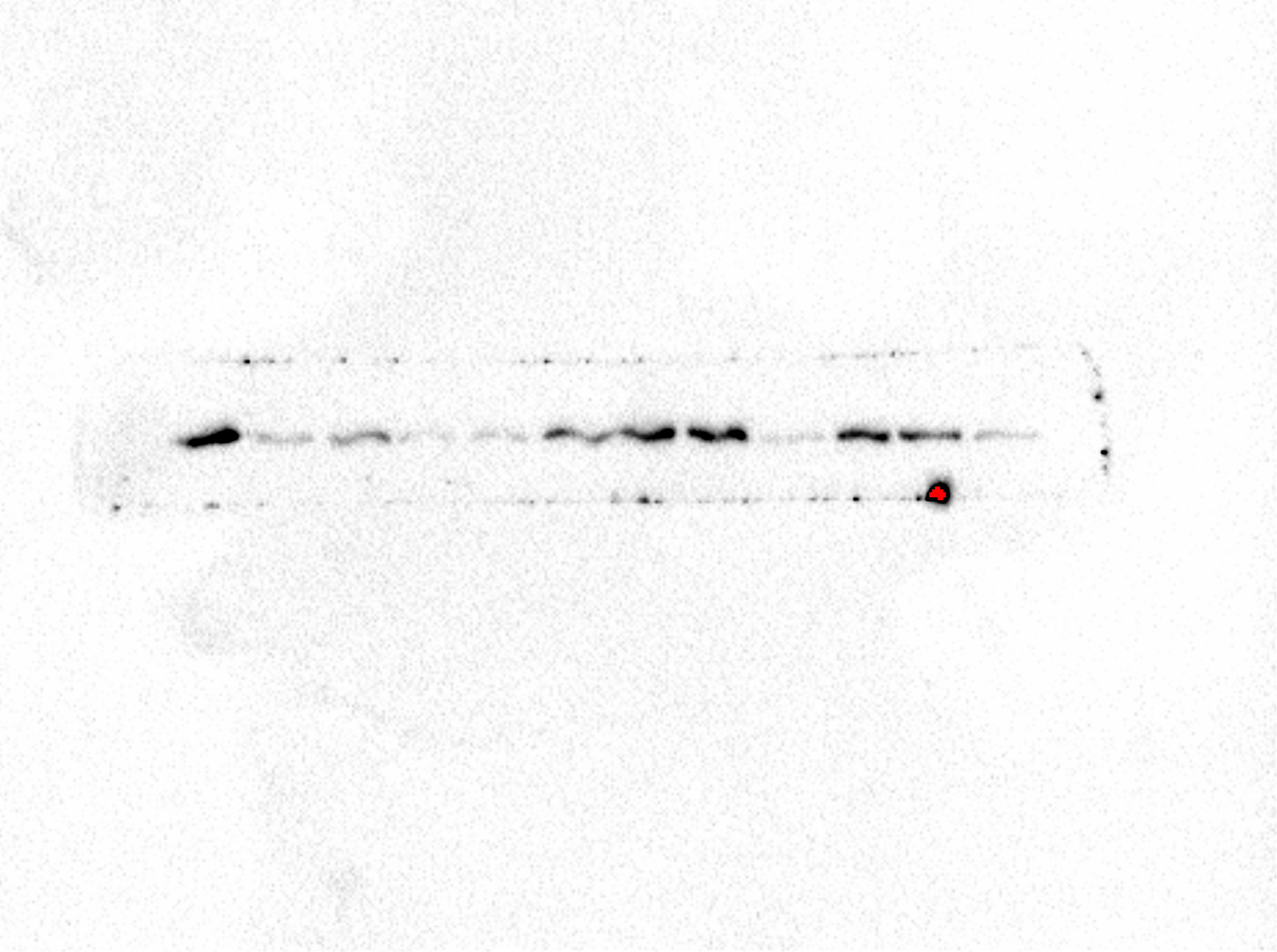

Supplement: Supplementary file 3 [file Data_Sheet_3.ZIP › Selivanova original blots single files/Membrane6-part3 (pAkt-Ser473).tif]
