# Supplementary material for: Thyroxine Induces Acute Relaxation of Rat Skeletal Muscle Arteries via Integrin αvβ3, ERK1/2 and Integrin-Linked Kinase
Source: Front Physiol. 2021 Sep 14;12:726354. doi: 10.3389/fphys.2021.726354 (PMC8477044; doi:10.3389/fphys.2021.726354)
Supplement: Supplementary file 4 [file Image_1.pdf]

Thyroxine induces acute relaxation of rat skeletal muscle arteries via integrin αvβ3, ERK1/2 and integrin-linked kinase

Ekaterina Selivanova, Dina Gaynullina, Olga Tarasova

Supplementary Materials

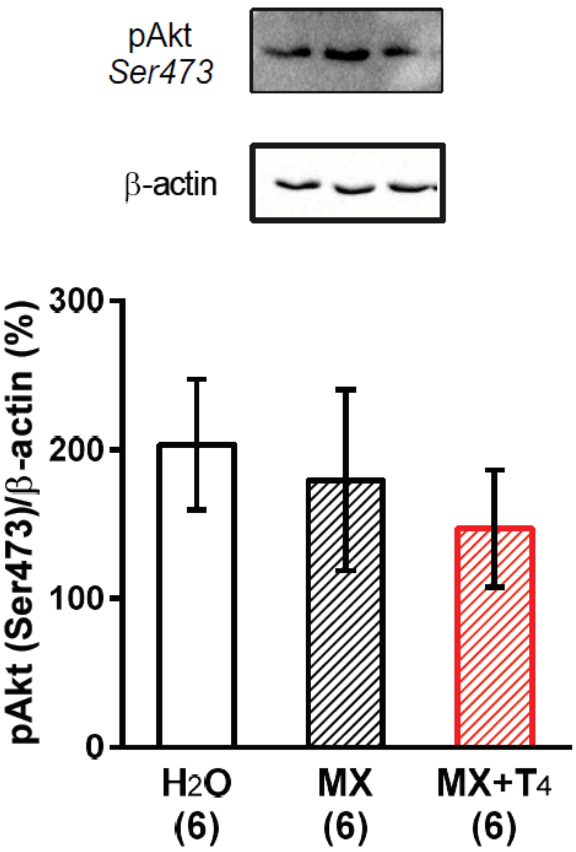

Supplementary Figure 1. T4 does not affect phospho-Akt (Ser473) content in sural arteries. The representative membrane parts are shown in the top. Data were normalized to β-actin level in the same sample and then the average value of two reference samples was taken as 100%. Number in parentheses represents the number of animals.
